# Supplementary material for: How reliable is metabarcoding for pollen identification? An evaluation of different taxonomic assignment strategies by cross-validation
Source: PeerJ. 2024 Jan 31;12:e16567. doi: 10.7717/peerj.16567 (PMC10838070; doi:10.7717/peerj.16567)
Supplement: Supplemental Information 1 [file peerj-12-16567-s001.pdf]

How reliable is metabarcoding for pollen identification? An evaluation of different taxonomic assignment strategies by cross-validation. : Supplements

Gilles San Martin Louis Hautier Dominique Mingeot Benjamin Dubois

20 September 2023 - 03:46

Contents

1 Introduction 2
1.1 Summary of the tested strategies . . . 3
1.2 Examples . . . 4

2 Description of the databases 8
2.1 Number of sequences in the databases & number of query sequences . . . 8
2.1.1 Number of sequences present in the original databases . . . 8
2.1.2 Number of query sequences blasted for each combination. . . . 8
2.2 Distribution of the alignment lengths . . . 9
2.3 Taxonomic coverage of the databases . . . 10
2.4 Missing taxonomic assignments . . . 12
2.4.1 % of sequences without any blast hits . . . 12
2.4.2 % of sequences with no taxonomic assignation by TopNPlus method . . . 13

3 % of correctly identified sequences for each taxonomic level 14
3.1 Descriptive barcharts . . . 14
3.1.1 All levels together . . . 14
3.1.2 % correct predictions at the species level . . . 16
3.1.3 % correct predictions at the genus level . . . 17
3.1.4 % correct predictions at the family level . . . 18
3.1.5 % correct predictions at the order level . . . 19
3.2 Comparisons . . . 20
3.2.1 Compare the 4 taxonomic assignation methods . . . 20
3.2.2 Compare 10 fold CV vs leaked CV & General vs Restricted . . . 22
3.2.3 Compare World vs Local database + ITS2 vs rbcL . . . 24

4 Identity and Consensus scores as predictors of the taxonomic assignation quality 27
4.1 Simple graphical representation (Single predictor binomial GLMs) . . . 27
4.1.1 On a World Database . . . 27
4.1.2 On a Local Database . . . 28
4.1.3 Distribution of the scores . . . 29
4.2 Separate multivariate binomial GLMs : `correct ID ~ Consensus * Identity` . . . 31
4.2.1 Model coefficients . . . 31
4.2.2 Distribution of the GLM predicted probabilities . . . 32
4.2.3 Graphical representation of predicted probability of correct ID . . . 33

4.2.4 Using classification trees to help choose a threshold . . . 34
4.3 Classification trees : predict if the ID is correct at each taxonomic level . . . 35
4.3.1 Species level - ITS2 - Local DB . . . 35
4.3.2 Species level - rbcL - Local DB . . . 35
4.3.3 Species level - ITS2 - World DB . . . 36
4.3.4 Species level - rbcL - World DB . . . 36
4.3.5 Genus level - ITS2 . . . 37
4.3.6 Genus level - rbcL . . . 37
4.4 Classification trees : Predict the best taxonomic level with identity and consensus scores . . . 38
4.4.1 ITS2 . . . 38
4.4.2 rbcL . . . 38
4.5 Reduction of prediction error when we apply exclusion rules based on the Classification trees 39

5 Compare ITS2 and rbcL for various taxonomic groups 41
5.1 % of correctly identified sequences for each Family . . . 41
5.1.1 Local species - Local database . . . 41
5.1.2 Local species - World database . . . 44
5.1.3 All species - World database . . . 45
5.2 % of correctly identified sequences for various genus of interest . . . 47
5.2.1 Fruit trees . . . 47
5.2.2 Brassicaceae . . . 53

6 Session Info 56

7 References 57

---

1 Introduction

The aim of this document is to provide an in depth analysis of the data used in the paper along with the full code to allow perfect reproducibility of our results. It contains the computations and graphs presented in the paper along with other approaches tested and not included in the main paper.

This document is available in a figshare archive (https://doi.org/10.6084/m9.figshare.23691579) with the R code and all the data necessary to reproduce our results.

1.1 Summary of the tested strategies

The aim of this paper is to evaluate the relative accuracy of taxonomic assignations when we chose different strategies based on blast. Please refer to the paper for details.

Our approach is based on cross-validation : we extract a random sample of sequences with known taxonomies from a reference database, apply various taxonomic assignment strategies and then compare the true taxon (eg `Taxon_true`, `Species_true`,or simply `Species`, . . .) with the taxon assigned (`Taxon_predicted`, `Species_predicted`) and compute the % of correctly predicted sequences (`Accuracy`) and in some circumstances other statistics (`Recall`, `Precision`, `F Score`).

We compute two statistics describing the quality of the match :

- `Identity score` (`Ident`) : Identity (%) corresponding to the best Bit score of the assigned taxon
- `Consensus score` (`Cons`) : % of sequences among the top N hits corresponding to the same taxon

Summary of the strategies tested and the factors that could affect the results : –> a total of 192 combinations for each taxonomic level

- 4 blast assignation `Method` :
  - `TopHit` : best blast hit (based on the Bit score)
  - `TopHitPlus` : best blast hit + in case of tied Bit scores, chose the one with the highest consensus score
  - `TopN` : taxon with the best consensus score among the top 10 hits
  - `TopNPlus` : taxon with the best consensus score computed after eliminating hits considered of lower quality based on various criteria (eg at least 97% identity at the species level, 90% at the genus level and 80% at the family level, etc. . .)
- 2 `Barcode` : full extraction from NCBI, keeping only Magnoliopsida and Pinopsida
  - `ITS2`
  - `rbcL`
- 3 Region of of the gene considered (`DB_gene`) :
  - `Restricted` : reference sequences trimmed to consider only the region amplified by one primer used in our study case
  - `General` : full length of the sequences present in the databases
  - `Restricted-General` : we blast the restricted sequences against the database with the full length sequences
- 2 species `Origin` :
  - `Local sp.` : species known to be present in the study area (Belgium) including crops, exotics, occasional species,. . . but excluding ornamental plants from gardens, parks,. . .
  - `Foreign sp.` : species not known from the area or planted in urbanized areas
- 2 database geographic area (`DB_area`) :
  - `Local DB` : reference database containing only the local species
  - `World DB` : reference database containing sequences of species from all around the World
- 2 cross validation strategy (`CV_method`) :
  - `10 Fold CV` : classical 10 fold cross validation : 10% of the sequences are blasted against the other 90% of the sequences, then the process is repeated –> this might tend to under-estimate the assignations accuracy because the "true" sequence is no more in the reference DB
  - `Leaked CV` : the sequences blasted are not removed from the reference DB –> this approach tests whether blast is able to match the true sequence when it is really present in the database –> this will tend to over-estimate the real accuracy of taxonomic assignations
- 4 taxonomic level (`Tax_level`) : we assign a taxon for each major taxonomic level : `Species`, `Genus`, `Family`, `Order`

1.2 Examples

Good example of the differences between the methods for 1 target sequence corresponding to *Scirpoides holoschoenus* (General + World DB).

Top 15 blast hits :

Species_true	Bit_score	Length	Identity	Family	Genus	Species
Scirpoides holoschoenus	1075	589	99.660	Cyperaceae	Scirpoides	Scirpoides holoschoenus
Scirpoides holoschoenus	704	607	88.138	Cyperaceae	Erioscirpus	Erioscirpus microstachyus
Scirpoides holoschoenus	699	608	87.993	Cyperaceae	Erioscirpus	Erioscirpus comosus
Scirpoides holoschoenus	682	608	87.500	Cyperaceae	Erioscirpus	Erioscirpus comosus
Scirpoides holoschoenus	651	595	86.891	Cyperaceae	Dracoscirpoides	Dracoscirpoides ficinioides
Scirpoides holoschoenus	630	597	86.265	Cyperaceae	Dracoscirpoides	Dracoscirpoides falsa
Scirpoides holoschoenus	630	597	86.265	Cyperaceae	Dracoscirpoides	Dracoscirpoides falsa
Scirpoides holoschoenus	549	568	84.507	Cyperaceae	Cyperus	Cyperus leucocephalus
Scirpoides holoschoenus	542	589	83.701	Cyperaceae	Cyperus	Cyperus trialatus
Scirpoides holoschoenus	529	570	83.860	Cyperaceae	Cyperus	Cyperus haspan
Scirpoides holoschoenus	525	571	83.713	Cyperaceae	Cyperus	Cyperus flaccidus
Scirpoides holoschoenus	520	616	82.630	Cyperaceae	Cyperus	Cyperus sp.
Scirpoides holoschoenus	520	609	82.594	Cyperaceae	Cyperus	Cyperus diffusus
Scirpoides holoschoenus	520	609	82.594	Cyperaceae	Cyperus	Cyperus diffusus
Scirpoides holoschoenus	518	569	83.480	Cyperaceae	Cyperus	Cyperus tenuispica

Results for the 4 assignation methods.

`Species` is the taxon assigned at Species level with its Maximum Identity score (`s_Ident`) and consensus score (`s_Cons`). The same information is provided for the Genus and Family taxonomic assignments. `TaxScore` is the best taxonomic level we should use for each target sequence and assignation method. A value of 7 means that the ID is correct up to the species level, 6 to the genus level, 5 to the family level, . . . , 1 to the kingdom level.

Method	Species	s_Ident	s_Cons	Genus	g_Ident	g_Cons	Family	f_Ident	f_Cons	TaxScore
TopHit	Scirpoides holoschoenus	99.660	10	Scirpoides	99.660	10	Cyperaceae	99.66	100	7
TopHitPlus	Scirpoides holoschoenus	99.660	10	Scirpoides	99.660	10	Cyperaceae	99.66	100	7
TopN	Erioscirpus comosus	87.993	20	Erioscirpus	88.138	30	Cyperaceae	99.66	100	5
TopNPlus	Scirpoides holoschoenus	99.660	100	Scirpoides	99.660	100	Cyperaceae	99.66	100	7

Best Bit score (`TopHit`) = Scirpoides holoschoenus with Identity = 99.7 but Consensus score of only 1/10 (10%)

Consensus top 10 approach (`TopN`) : Erioscirpus comosus is chosen because consensus = 2/10, even if identity is only 88%.

Note that at the genus level a different genus could have been chosen. For example if we used the top 15 hits, the chosen species would have been Erioscirpus comosus but the chosen genus would have been Cyperus because Cyperus would have a higher consensus score based on the top 15 hits. With the TopHit approches, the species and genus are always congruent but with topN it is not necessarily the case.

For TopNPlus, the Consensus score is 100% at species and Genus level because at species level we discard all hits with identity < 97% and at genus level all hits with identity <90% (so there is only the first line left). At family level we keep all first 10 hits because their Identity is > 80% but they are all from the same family so the Consensus score is 100% anyway.

There are also probably errors in the reference database... For example the following IDs have a "wrong" Family level identification while the identity score is 100% and the consensus score is also 100% –> the taxonomy proposed in the database for the target sequence is probably wrong.

This represent however only a very small fraction of all sequences. It also means that in real life, these sequences would probably be assigned to the right taxon even if in the cross validation these assignations are considered as wrong.

```
##  [1] "AJ245942.1" "AJ439911.1" "AJ439912.1" "AJ439947.1" "AJ439955.1" "AJ439983.1" "AJ490885.1"
##  [8] "AJ490921.1" "AJ490926.1" "EF590505.1" "FR865163.1" "HE963559.1" "HG417045.1" "JN191139.1"
## [15] "KF997379.1" "KJ747432.1" "KJ747433.1" "KJ747440.1" "KJ747442.1" "KJ747444.1" "KJ747447.1"
## [22] "KJ747463.1" "KJ747464.1" "KJ747466.1" "KJ747472.1" "KJ747473.1" "KJ747474.1" "KJ747475.1"
## [29] "KJ747489.1" "KJ747499.1" "KJ747506.1" "KJ747507.1" "KJ747509.1" "KT960119.1" "KU748337.1"
## [36] "KU936076.1" "KU991227.1" "KX344593.1" "KX344714.1" "MG215740.1" "MG225716.1" "MG235449.1"
## [43] "MG246977.1" "MN206545.1"
```

```
## # A tibble: 2 x 39
##   Label                 Barcode CV_method  DB_area  DB_gene Origin    Wild  Fold  Method
##   <chr>                 <chr>   <chr>      <chr>    <chr>   <chr>     <chr> <chr> <chr>
## 1 CV_ITS2_general_world ITS2    10 Fold CV World DB General Local sp. Wild  F04   TopHitPlus
## 2 CV_ITS2_general_world ITS2    10 Fold CV World DB General Local sp. Wild  F10   TopHitPlus
##   TaxID_query Order_true     Family_true   Genus_true   Species_true      Order          o_Bit
##   <chr>       <chr>          <chr>         <chr>        <chr>             <chr>          <dbl>
## 1 MG215740.1  Poales         Cyperaceae    Rhynchospora Rhynchospora alba Ericales        588
## 2 MG235449.1  Caryophyllales Amaranthaceae Polycnemum   Polycnemum majus  Caryophyllales   616
##   o_Length o_Ident o_Cons OrderOK Family         f_Bit f_Length f_Ident f_Cons FamilyOK
##      <int>   <dbl>  <dbl>   <dbl> <chr>          <dbl>    <int>   <dbl>  <dbl>    <dbl>
## 1      318     100    100       0 Balsaminaceae    588      318     100    100        0
## 2      333     100    100       1 Chenopodiaceae   616      333     100    100        0
##   Genus     g_Bit g_Length g_Ident g_Cons GenusOK Species            s_Bit s_Length s_Ident
##   <chr>     <dbl>    <int>   <dbl>  <dbl>   <dbl> <chr>              <dbl>    <int>   <dbl>
## 1 Impatiens   588      318     100    100       0 Impatiens capensis   588      318     100
## 2 Salsola     616      333     100     90       0 Salsola tragus       616      333     100
##   s_Cons SpeciesOK TaxScore
##    <dbl>     <dbl>    <dbl>
## 1     40         0        3
## 2     60         0        4
```

A few examples of these plausible database errors :

TaxID_query	Family_true	Species_true	Bit_score	Identity	Family	Species
AJ245942.1	Sapindaceae	Acer buergerianum	193	100.000	Poaceae	Setaria italica
AJ245942.1	Sapindaceae	Acer buergerianum	193	100.000	Poaceae	Zea mays
AJ245942.1	Sapindaceae	Acer buergerianum	193	100.000	Poaceae	Hygroryza aristata
AJ245942.1	Sapindaceae	Acer buergerianum	193	100.000	Poaceae	Bothriochloa ischaemum
AJ245942.1	Sapindaceae	Acer buergerianum	193	100.000	Poaceae	Cenchrus flaccidus
AJ245942.1	Sapindaceae	Acer buergerianum	193	100.000	Poaceae	Cenchrus centrasiaticus
AJ245942.1	Sapindaceae	Acer buergerianum	193	100.000	Poaceae	Tripsacum laxum
AJ245942.1	Sapindaceae	Acer buergerianum	193	100.000	Poaceae	Ehrharta erecta
AJ245942.1	Sapindaceae	Acer buergerianum	193	100.000	Poaceae	Coix aquatica
AJ245942.1	Sapindaceae	Acer buergerianum	193	100.000	Poaceae	Sarga versicolor
AJ439911.1	Gesneriaceae	Sinningia reitzii	294	100.000	Lamiaceae	Hyssopus officinalis
AJ439911.1	Gesneriaceae	Sinningia reitzii	294	100.000	Lamiaceae	Salvia glutinosa
AJ439911.1	Gesneriaceae	Sinningia reitzii	294	100.000	Lamiaceae	Salvia rutilans
AJ439911.1	Gesneriaceae	Sinningia reitzii	294	100.000	Lamiaceae	Horminum pyrenaicum
AJ439911.1	Gesneriaceae	Sinningia reitzii	294	100.000	Lamiaceae	Salvia glutinosa
AJ439911.1	Gesneriaceae	Sinningia reitzii	294	100.000	Lamiaceae	Satureja thymbra
AJ439911.1	Gesneriaceae	Sinningia reitzii	294	100.000	Lamiaceae	Lavandula stoechas
AJ439911.1	Gesneriaceae	Sinningia reitzii	294	100.000	Lamiaceae	Satureja montana
AJ439911.1	Gesneriaceae	Sinningia reitzii	294	100.000	Lamiaceae	Lavandula angustifolia
AJ439911.1	Gesneriaceae	Sinningia reitzii	294	100.000	Lamiaceae	Agastache rugosa
AJ439912.1	Gesneriaceae	Sinningia brasiliensis	294	100.000	Lamiaceae	Hyssopus officinalis
AJ439912.1	Gesneriaceae	Sinningia brasiliensis	294	100.000	Lamiaceae	Salvia glutinosa
AJ439912.1	Gesneriaceae	Sinningia brasiliensis	294	100.000	Lamiaceae	Salvia rutilans
AJ439912.1	Gesneriaceae	Sinningia brasiliensis	294	100.000	Lamiaceae	Horminum pyrenaicum
AJ439912.1	Gesneriaceae	Sinningia brasiliensis	294	100.000	Lamiaceae	Salvia glutinosa
AJ439912.1	Gesneriaceae	Sinningia brasiliensis	294	100.000	Lamiaceae	Satureja thymbra
AJ439912.1	Gesneriaceae	Sinningia brasiliensis	294	100.000	Lamiaceae	Lavandula stoechas
AJ439912.1	Gesneriaceae	Sinningia brasiliensis	294	100.000	Lamiaceae	Horminum pyrenaicum

TaxID_query	Family_true	Species_true	Bit_score	Identity	Family	Species
AJ439912.1	Gesneriaceae	Sinningia brasiliensis	294	100.000	Lamiaceae	Lavandula angustifolia
AJ439912.1	Gesneriaceae	Sinningia brasiliensis	294	100.000	Lamiaceae	Agastache rugosa
AJ439947.1	Gesneriaceae	Sinningia elatior	294	100.000	Lamiaceae	Hyssopus officinalis
AJ439947.1	Gesneriaceae	Sinningia elatior	294	100.000	Lamiaceae	Salvia glutinosa
AJ439947.1	Gesneriaceae	Sinningia elatior	294	100.000	Lamiaceae	Salvia rutilans
AJ439947.1	Gesneriaceae	Sinningia elatior	294	100.000	Lamiaceae	Horminum pyrenaicum
AJ439947.1	Gesneriaceae	Sinningia elatior	294	100.000	Lamiaceae	Salvia glutinosa
AJ439947.1	Gesneriaceae	Sinningia elatior	294	100.000	Lamiaceae	Satureja thymbra
AJ439947.1	Gesneriaceae	Sinningia elatior	294	100.000	Lamiaceae	Lavandula stoechas
AJ439947.1	Gesneriaceae	Sinningia elatior	294	100.000	Lamiaceae	Satureja montana
AJ439947.1	Gesneriaceae	Sinningia elatior	294	100.000	Lamiaceae	Lavandula angustifolia
AJ439947.1	Gesneriaceae	Sinningia elatior	294	100.000	Lamiaceae	Agastache rugosa
AJ439955.1	Gesneriaceae	Sinningia curtiflora	294	100.000	Lamiaceae	Hyssopus officinalis
AJ439955.1	Gesneriaceae	Sinningia curtiflora	294	100.000	Lamiaceae	Salvia glutinosa
AJ439955.1	Gesneriaceae	Sinningia curtiflora	294	100.000	Lamiaceae	Salvia rutilans
AJ439955.1	Gesneriaceae	Sinningia curtiflora	294	100.000	Lamiaceae	Horminum pyrenaicum
AJ439955.1	Gesneriaceae	Sinningia curtiflora	294	100.000	Lamiaceae	Salvia glutinosa
AJ439955.1	Gesneriaceae	Sinningia curtiflora	294	100.000	Lamiaceae	Lavandula stoechas
AJ439955.1	Gesneriaceae	Sinningia curtiflora	294	100.000	Lamiaceae	Horminum pyrenaicum
AJ439955.1	Gesneriaceae	Sinningia curtiflora	294	100.000	Lamiaceae	Satureja montana
AJ439955.1	Gesneriaceae	Sinningia curtiflora	294	100.000	Lamiaceae	Lavandula angustifolia
AJ439955.1	Gesneriaceae	Sinningia curtiflora	294	100.000	Lamiaceae	Agastache rugosa
AJ439983.1	Gesneriaceae	Gloxinia erinoides	294	100.000	Lamiaceae	Hyssopus officinalis
AJ439983.1	Gesneriaceae	Gloxinia erinoides	294	100.000	Lamiaceae	Salvia glutinosa
AJ439983.1	Gesneriaceae	Gloxinia erinoides	294	100.000	Lamiaceae	Salvia rutilans
AJ439983.1	Gesneriaceae	Gloxinia erinoides	294	100.000	Lamiaceae	Horminum pyrenaicum
AJ439983.1	Gesneriaceae	Gloxinia erinoides	294	100.000	Lamiaceae	Salvia glutinosa
AJ439983.1	Gesneriaceae	Gloxinia erinoides	294	100.000	Lamiaceae	Satureja thymbra
AJ439983.1	Gesneriaceae	Gloxinia erinoides	294	100.000	Lamiaceae	Lavandula stoechas
AJ439983.1	Gesneriaceae	Gloxinia erinoides	294	100.000	Lamiaceae	Horminum pyrenaicum
AJ439983.1	Gesneriaceae	Gloxinia erinoides	294	100.000	Lamiaceae	Satureja montana
AJ439983.1	Gesneriaceae	Gloxinia erinoides	294	100.000	Lamiaceae	Agastache rugosa
AJ490885.1	Scrophulariaceae	Verbascum speciosum	289	100.000	Lamiaceae	Salvia hispanica
AJ490885.1	Scrophulariaceae	Verbascum speciosum	289	100.000	Lamiaceae	Salvia canariensis
AJ490885.1	Scrophulariaceae	Verbascum speciosum	289	100.000	Lamiaceae	Salvia bucharica
AJ490885.1	Scrophulariaceae	Verbascum speciosum	289	100.000	Lamiaceae	Salvia officinalis
AJ490885.1	Scrophulariaceae	Verbascum speciosum	289	100.000	Lamiaceae	Lavandula latifolia
AJ490885.1	Scrophulariaceae	Verbascum speciosum	289	100.000	Lamiaceae	Salvia argentea
AJ490885.1	Scrophulariaceae	Verbascum speciosum	289	100.000	Lamiaceae	Salvia viridis
AJ490885.1	Scrophulariaceae	Verbascum speciosum	289	100.000	Lamiaceae	Salvia nemorosa
AJ490885.1	Scrophulariaceae	Verbascum speciosum	289	100.000	Lamiaceae	Teucrium scorodonia
AJ490885.1	Scrophulariaceae	Verbascum speciosum	289	100.000	Lamiaceae	Teucrium flavum
KT960119.1	Cyperaceae	Carex aquatilis	573	100.000	Brassicaceae	Draba arctogena
KT960119.1	Cyperaceae	Carex aquatilis	573	100.000	Brassicaceae	Draba puvirnituqii
KT960119.1	Cyperaceae	Carex aquatilis	573	100.000	Brassicaceae	Draba cana
KT960119.1	Cyperaceae	Carex aquatilis	573	100.000	Brassicaceae	Draba oblongata
KT960119.1	Cyperaceae	Carex aquatilis	573	100.000	Brassicaceae	Draba arctica
KT960119.1	Cyperaceae	Carex aquatilis	573	100.000	Brassicaceae	Draba subcapitata
KT960119.1	Cyperaceae	Carex aquatilis	573	100.000	Brassicaceae	Draba corymbosa
KT960119.1	Cyperaceae	Carex aquatilis	573	100.000	Brassicaceae	Draba alpina
KT960119.1	Cyperaceae	Carex aquatilis	569	99.677	Brassicaceae	Draba lactea
KT960119.1	Cyperaceae	Carex aquatilis	569	99.677	Brassicaceae	Draba lactea
MG215740.1	Cyperaceae	Rhynchospora alba	588	100.000	Balsaminaceae	Impatiens ecornuta
MG215740.1	Cyperaceae	Rhynchospora alba	588	100.000	Balsaminaceae	Impatiens noli-tangere
MG215740.1	Cyperaceae	Rhynchospora alba	588	100.000	Balsaminaceae	Impatiens capensis
MG215740.1	Cyperaceae	Rhynchospora alba	582	99.686	Balsaminaceae	Impatiens capensis
MG215740.1	Cyperaceae	Rhynchospora alba	582	99.686	Balsaminaceae	Impatiens ecornuta
MG215740.1	Cyperaceae	Rhynchospora alba	582	99.686	Balsaminaceae	Impatiens capensis
MG215740.1	Cyperaceae	Rhynchospora alba	582	99.686	Balsaminaceae	Impatiens pallida
MG215740.1	Cyperaceae	Rhynchospora alba	580	99.685	Balsaminaceae	Impatiens noli-tangere
MG215740.1	Cyperaceae	Rhynchospora alba	529	99.317	Balsaminaceae	Impatiens ecornuta
MG215740.1	Cyperaceae	Rhynchospora alba	521	100.000	Balsaminaceae	Impatiens capensis
MG235449.1	Amaranthaceae	Polycnemum majus	616	100.000	Chenopodiaceae	Salsola tragus
MG235449.1	Amaranthaceae	Polycnemum majus	612	99.700	Chenopodiaceae	Salsola tragus
MG235449.1	Amaranthaceae	Polycnemum majus	582	98.204	Chenopodiaceae	Salsola collina
MG235449.1	Amaranthaceae	Polycnemum majus	582	98.204	Chenopodiaceae	Salsola collina
MG235449.1	Amaranthaceae	Polycnemum majus	577	97.904	Chenopodiaceae	Kali turgidum
MG235449.1	Amaranthaceae	Polycnemum majus	549	100.000	Chenopodiaceae	Salsola tragus
MG235449.1	Amaranthaceae	Polycnemum majus	544	100.000	Chenopodiaceae	Salsola tragus
MG235449.1	Amaranthaceae	Polycnemum majus	542	99.331	Chenopodiaceae	Salsola tragus
MG235449.1	Amaranthaceae	Polycnemum majus	542	99.331	Chenopodiaceae	Salsola tragus

TaxID_query	Family_true	Species_true	Bit_score	Identity	Family	Species
MG235449.1	Amaranthaceae	Polycnemum majus	542	99.331	Chenopodiaceae	Salsola komarovii

2 Description of the databases

2.1 Number of sequences in the databases & number of query sequences

2.1.1 Number of sequences present in the original databases

NB : these counts contains also non Magniolopsida/Pinopsida species and also a few sequences that have been removed form the blasted sequences because the identification was not precise to the species level.

Barcode	DB_gene	n
ITS2	General	171754
ITS2	Restricted	22010
rbcL	General	134321
rbcL	Restricted	14714

Barcode	DB_gene	Origin	n
ITS2	General	Foreign sp.	154450
ITS2	General	Local sp.	17304
ITS2	Restricted	Foreign sp.	20424
ITS2	Restricted	Local sp.	1586
rbcL	General	Foreign sp.	117839
rbcL	General	Local sp.	16482
rbcL	Restricted	Foreign sp.	13291
rbcL	Restricted	Local sp.	1423

2.1.2 Number of query sequences blasted for each combination.

From the available sequences we selected a subset to be blasted (query sequences) containing all local species and ~10% of the foreign species from all databases. We filtered out the species that were not Magnioliopsida or Pinopsida and which were not identified unambiguously to the species level.

NB : the sequences used for blast are identical between the Local DB and World DB because we wanted to see what happens when you blast foreign species against a local database

DB_gene	Barcode	DB_area	Nb_seq
General	ITS2	Local DB	31922
General	ITS2	World DB	31922
General	rbcL	Local DB	25562
General	rbcL	World DB	25562
Restr-General	ITS2	Local DB	4938
Restr-General	ITS2	World DB	4938
Restr-General	rbcL	Local DB	3701
Restr-General	rbcL	World DB	3701
Restricted	ITS2	Local DB	4938
Restricted	ITS2	World DB	4938
Restricted	rbcL	Local DB	3701
Restricted	rbcL	World DB	3701

Same table with the detail of the relative frequency of Local and Foreign species among the blasted sequences (we show only the "World DB" results which are identical to the "Local DB" results)

DB_gene	Barcode	Origin	Nb_seq
General	ITS2	Foreign sp.	14691
General	ITS2	Local sp.	17231
General	rbcL	Foreign sp.	9941
General	rbcL	Local sp.	15621
Restr-General	ITS2	Foreign sp.	3365
Restr-General	ITS2	Local sp.	1573
Restr-General	rbcL	Foreign sp.	2307
Restr-General	rbcL	Local sp.	1394
Restricted	ITS2	Foreign sp.	3365
Restricted	ITS2	Local sp.	1573
Restricted	rbcL	Foreign sp.	2307
Restricted	rbcL	Local sp.	1394

2.2 Distribution of the alignment lengths

NB : we use a log10 scale and show the results only for World DB & Leaked CV & TopHitPlus

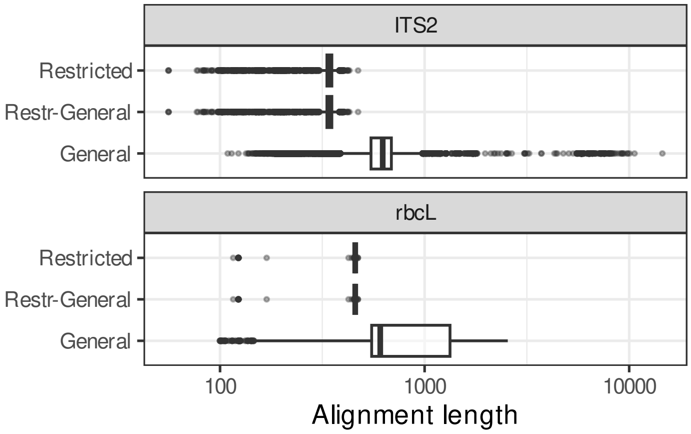

Figure 1:

Quantiles for the length of the alignment :

Barcode	DB_gene	min	q5	q10	q25	median	q75	q90	q95	max
ITS2	General	109	224	198	547	624	688	754	806	14531
ITS2	Restr-General	56	155	103	333	343	352	367	374	473
ITS2	Restricted	56	155	103	333	343	352	367	374	473
rbcL	General	100	484	174	551	607	1331	1428	1434	2550
rbcL	Restr-General	116	458	458	458	458	458	458	458	472
rbcL	Restricted	116	458	458	458	458	458	458	458	472

2.3 Taxonomic coverage of the databases

Taxonomic coverage at the species level = which proportion of the Belgian flora targeted in our study case are present in the reference databases at various taxonomic levels ?

Not easy to compute because of synonyms and potential differences of nomenclature between the Belgian species database and the NCBI nomenclature. –> estimates provided here are minimum.

For the species level only, we have added the species matched with synonyms in addition to the usual species names.

- `Ntot` = total number of species in the Belgian list
- `N` = number of these species found in the reference databases (+ synonyms)
- `Pct` = % of the species covered by the database

NB : "Wild" species are species reproducing in the wild (including naturalized/invasive species). "Introduced" are cultivated species (crops) or species occasionally observed in Belgium (occasional imports).

DB_gene	Wild	Barcode	Label	Ntot	N	Pct
General	Introduced	ITS2	ITS2_General	2026	1460	72.1
General	Introduced	rbcL	rbcL_General	2026	1350	66.6
General	Wild	ITS2	ITS2_General	1766	1433	81.1
General	Wild	rbcL	rbcL_General	1766	1555	88.1
Restricted	Introduced	ITS2	ITS2_Restricted	2026	408	20.1
Restricted	Introduced	rbcL	rbcL_Restricted	2026	613	30.3
Restricted	Wild	ITS2	ITS2_Restricted	1766	389	22.0
Restricted	Wild	rbcL	rbcL_Restricted	1766	540	30.6

Same computation but wild and introduced species pooled

DB_gene	Barcode	Label	Ntot	N	Pct
General	ITS2	ITS2_General	3792	2893	76.3
General	rbcL	rbcL_General	3792	2905	76.6
Restricted	ITS2	ITS2_Restricted	3792	797	21.0
Restricted	rbcL	rbcL_Restricted	3792	1153	30.4

Taxonomic coverage at the genus level

DB_gene	Wild	Barcode	Label	Ntot	N	Pct
General	Introduced	ITS2	ITS2_General	535	477	89.159
General	Introduced	rbcL	rbcL_General	535	478	89.346
General	Wild	ITS2	ITS2_General	651	614	94.316
General	Wild	rbcL	rbcL_General	651	632	97.081
Restricted	Introduced	ITS2	ITS2_Restricted	535	231	43.178
Restricted	Introduced	rbcL	rbcL_Restricted	535	339	63.364
Restricted	Wild	ITS2	ITS2_Restricted	651	395	60.676
Restricted	Wild	rbcL	rbcL_Restricted	651	452	69.432

Wild and introduced species pooled

DB_gene	Barcode	Label	Ntot	N	Pct
General	ITS2	ITS2_General	1186	1091	92.0
General	rbcL	rbcL_General	1186	1110	93.6
Restricted	ITS2	ITS2_Restricted	1186	626	52.8
Restricted	rbcL	rbcL_Restricted	1186	791	66.7

List of genus absent for ITS2 or rbcL.

Missing genus present in the wild –> several of them are probably present in the sequence database under a different name (Betonica, Cruciata, Vulpia, Hepatica,…). Most of these genus are very rare.

```
##  [1] "Anisantha"      "Athyrium"       "Betonica"       "Bromopsis"      "Calla"
##  [6] "Cardaminopsis"  "Centunculus"    "Ceratochloa"    "Crithmum"       "Cruciata"
## [11] "Crypsis"        "Cystopteris"    "Dichoropetalum" "Epipogium"      "Foeniculum"
## [16] "Genistella"     "Groenlandia"    "Gymnocarpium"   "Hammarbya"      "Hepatica"
## [21] "Hordelymus"     "Hyacinthoides"  "Hymenophyllum"  "Lamiastrum"     "Marrubium"
## [26] "Matteuccia"     "Monotropa"      "Mycelis"        "Nardurus"       "Onoclea"
## [31] "Orlaya"         "Parthenocissus" "Poterium"       "Schedonorus"    "Spartina"
## [36] "Spirodela"      "Struthiopteris" "Tamus"          "Telekia"        "Trichomanes"
## [41] "Vulpia"
```

Missing genus for occasional/introduced plants

```
##  [1] "Aconogonon"      "Adenostyles"     "Alonsoa"         "Amberboa"        "Ammobium"
##  [6] "Amphiachyris"    "Anisocampium"    "Anoda"           "Asarina"         "Asteriscus"
## [11] "Asterolinon"     "Attalea"         "Austrodanthonia" "Bifora"          "Biserrula"
## [16] "Blumenbachia"    "Boreava"         "Brachyachne"     "Brachycome"      "Calotis"
## [21] "Catananche"      "Cerinthe"        "Choisya"         "Cordylocarpus"   "Crithopsis"
## [26] "Crucianella"     "Danae"           "Darmera"         "Echinopsis"      "Ehrharta"
## [31] "Eichhornia"      "Einadia"         "Ellisiophyllum"  "Emex"            "Enarthrocarpus"
## [36] "Eremopoa"        "Erinus"          "Escallonia"      "Gutenbergia"     "Hebe"
## [41] "Heteranthemis"   "Hyacinthus"      "Hystrix"         "Ipheion"         "Isotoma"
## [46] "Kitaibelia"      "Lallemantia"     "Lepyrodiclis"    "Leucophyta"      "Lopezia"
## [51] "Lysichiton"      "Malope"          "Micropyrum"      "Nectaroscordum"  "Nonea"
## [56] "Opopanax"        "Pentaphylloides" "Perovskia"       "Phoenix"         "Pholiurus"
## [61] "Phuopsis"        "Physostegia"     "Podolepis"       "Pontederia"      "Ptychotis"
## [66] "Puschkinia"      "Rhagadiolus"     "Ridolfia"        "Rosmarinus"      "Ruscus"
## [71] "Sasaella"        "Schmidtia"       "Scorpiurus"      "Sibbaldiopsis"   "Skimmia"
## [76] "Smilacina"       "Stuartina"       "Taeniatherum"    "Tinantia"        "Tordylium"
## [81] "Tradescantia"    "Tricyrtis"       "Triteleia"       "Vaccaria"        "Waldsteinia"
```

2.4 Missing taxonomic assignments

2.4.1 % of sequences without any blast hits

For a few target sequences, blast didn't return any results (when E value >10 by default).

This is more frequent for :

- ITS2 : because rbcL is less specific, it is easier to find alternative matching sequences
- Foreign species when blasted against a Data Base restricted to the Local fauna (as expected) but this represents generally between 1 and 2 % of the sequences.

Note that for the Leaked CV approach and Local DB, foreign species have similar values compared to the true 10 fold CV because their sequences are never present in the Local Data Bases.

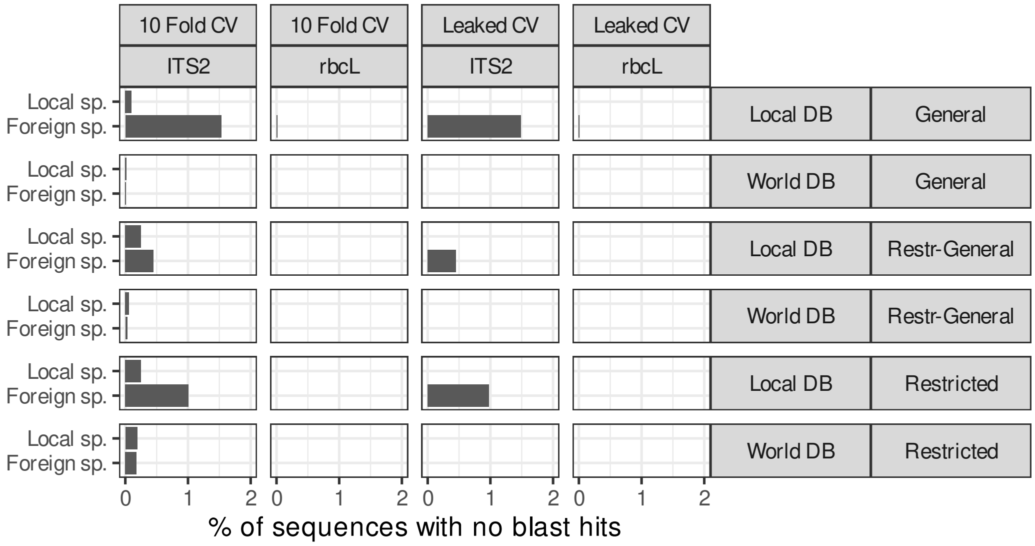

Figure 2:

2.4.2 % of sequences with no taxonomic assignation by TopNPlus method

% of sequences for which TopNPlus found no match either because blast didn't find any match (rarely) or because none of the top 10 blast matches passed the TopNPlus quality control (eg at least 97% identity at species level etc. . .).

As expected we have mostly no matches when we blast a Foreign species on a local Database. However this is true mainly for ITS2. This means that for rbcL TopNPlus finds some hits anyways even if they are wrong.

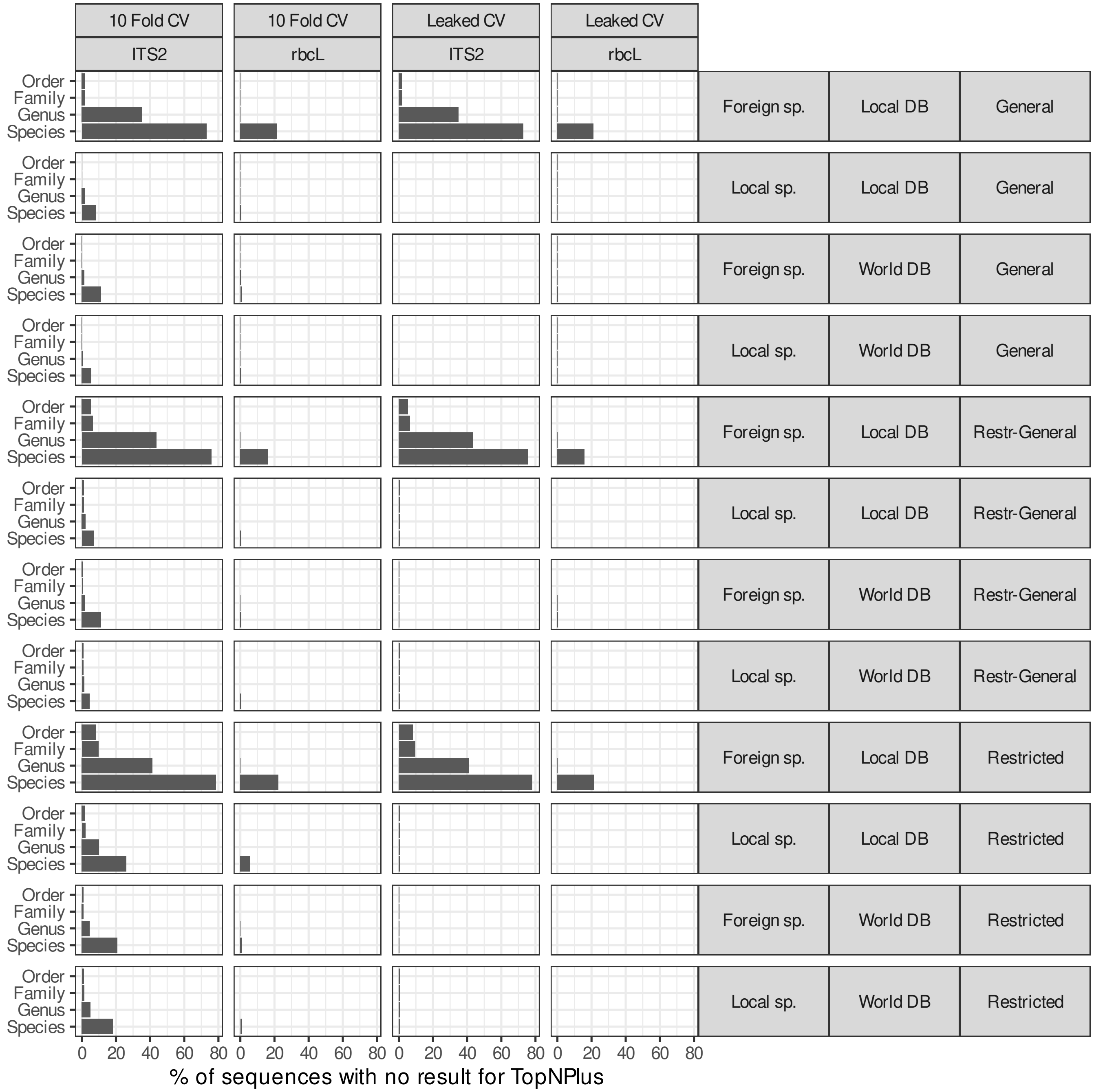

Figure 3:

3 % of correctly identified sequences for each taxonomic level

3.1 Descriptive barcharts

3.1.1 All levels together

NB : the following graph shows all results together. It is quite overloaded with information –> the same data will be shown from different and more simple points of view in the next pages.

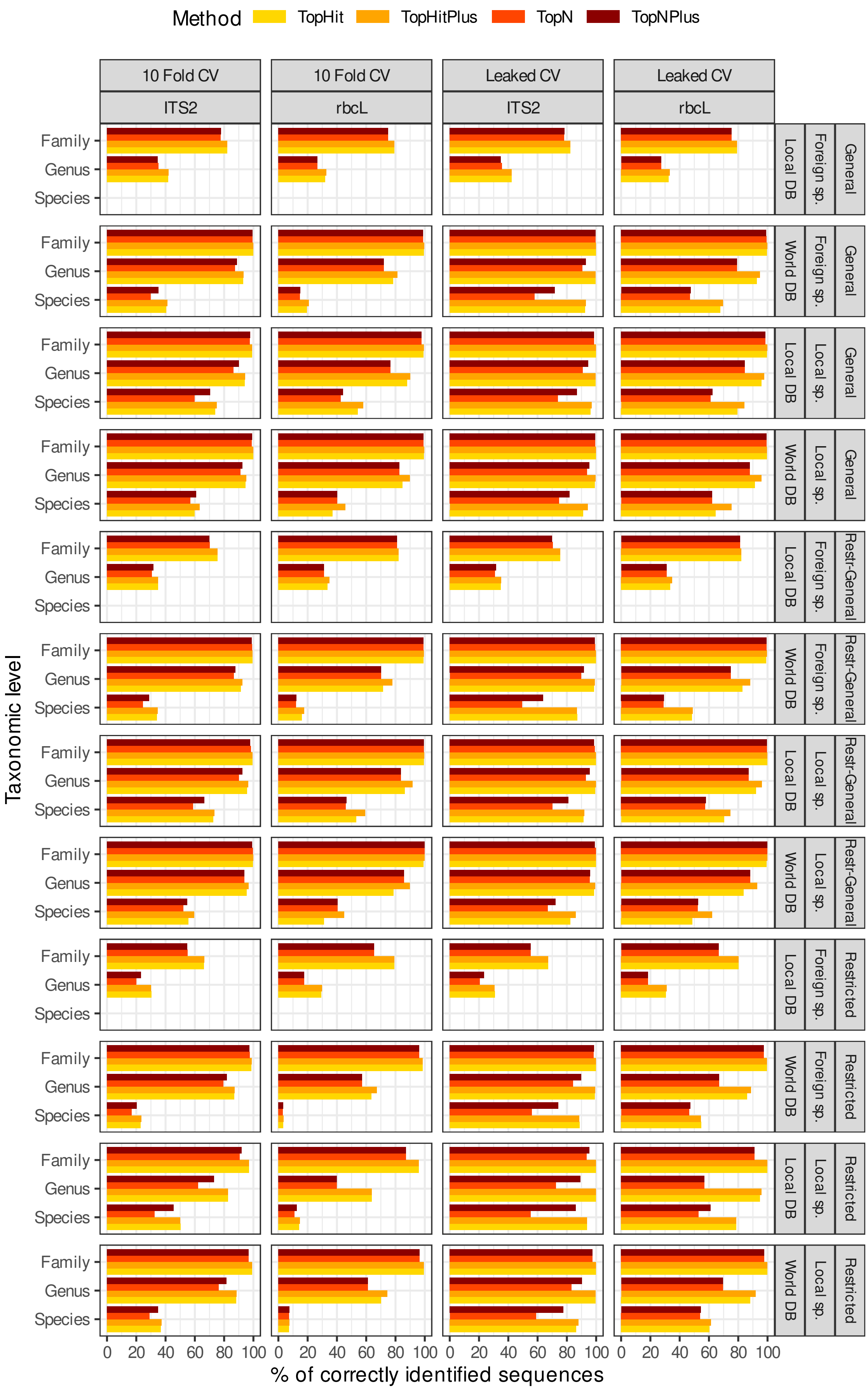

Method
TopHit
TopHitPlus
TopN
TopNPlus
10 Fold CV
Leaked CV
ITS2
rbcL
Family
Genus
Species
Local DB
World DB
Foreign sp.
Local sp.
General
Restr-General
Restricted
Taxonomic level
0 20 40 60 80 100
% of correctly identified sequences

Figure 4:

3.1.2 % correct predictions at the species level

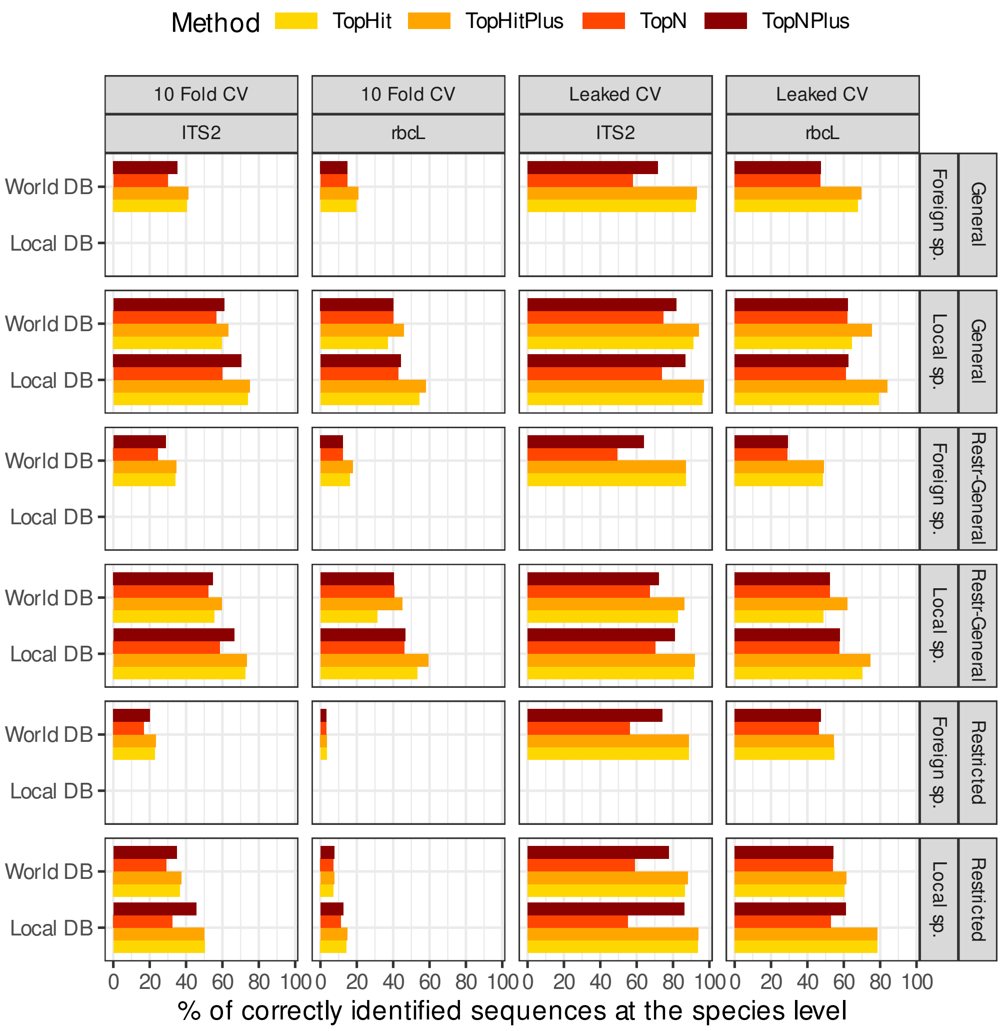

Figure 5:

3.1.3 % correct predictions at the genus level

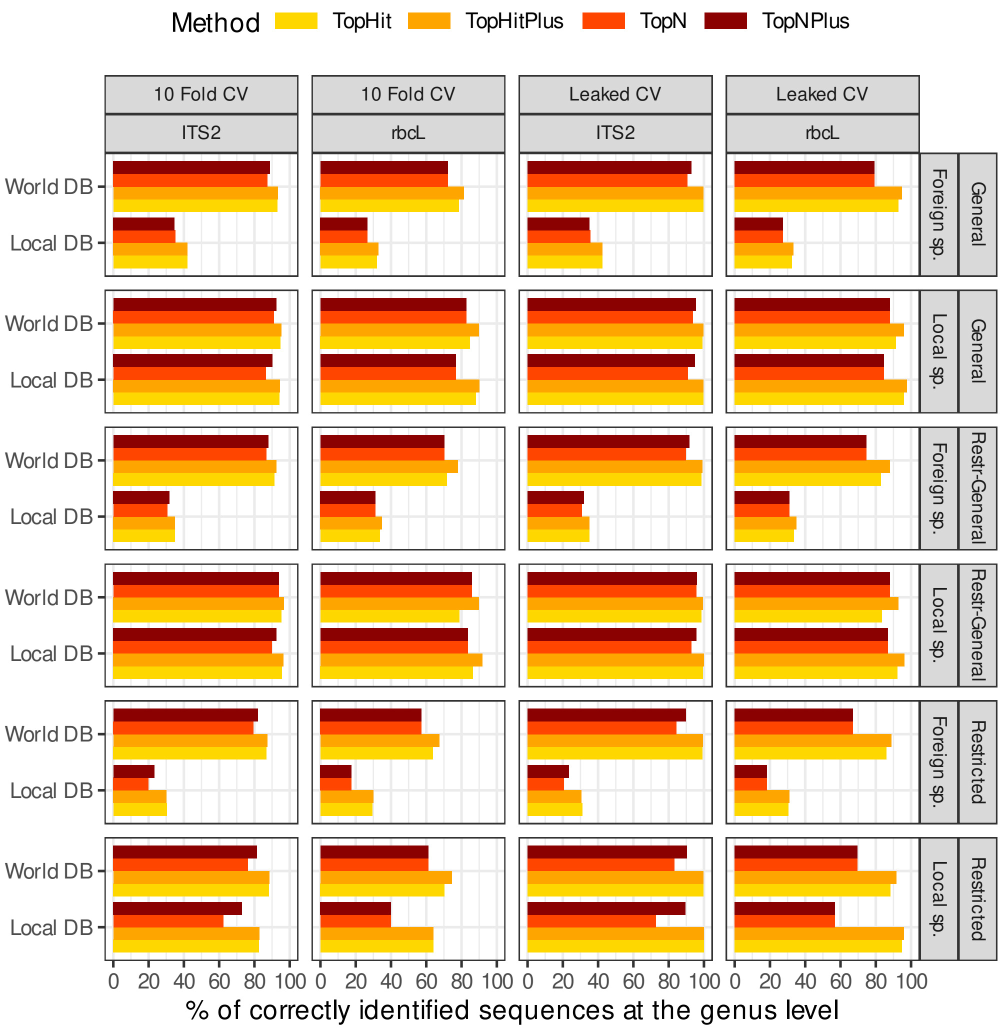

Figure 6:

3.1.4 % correct predictions at the family level

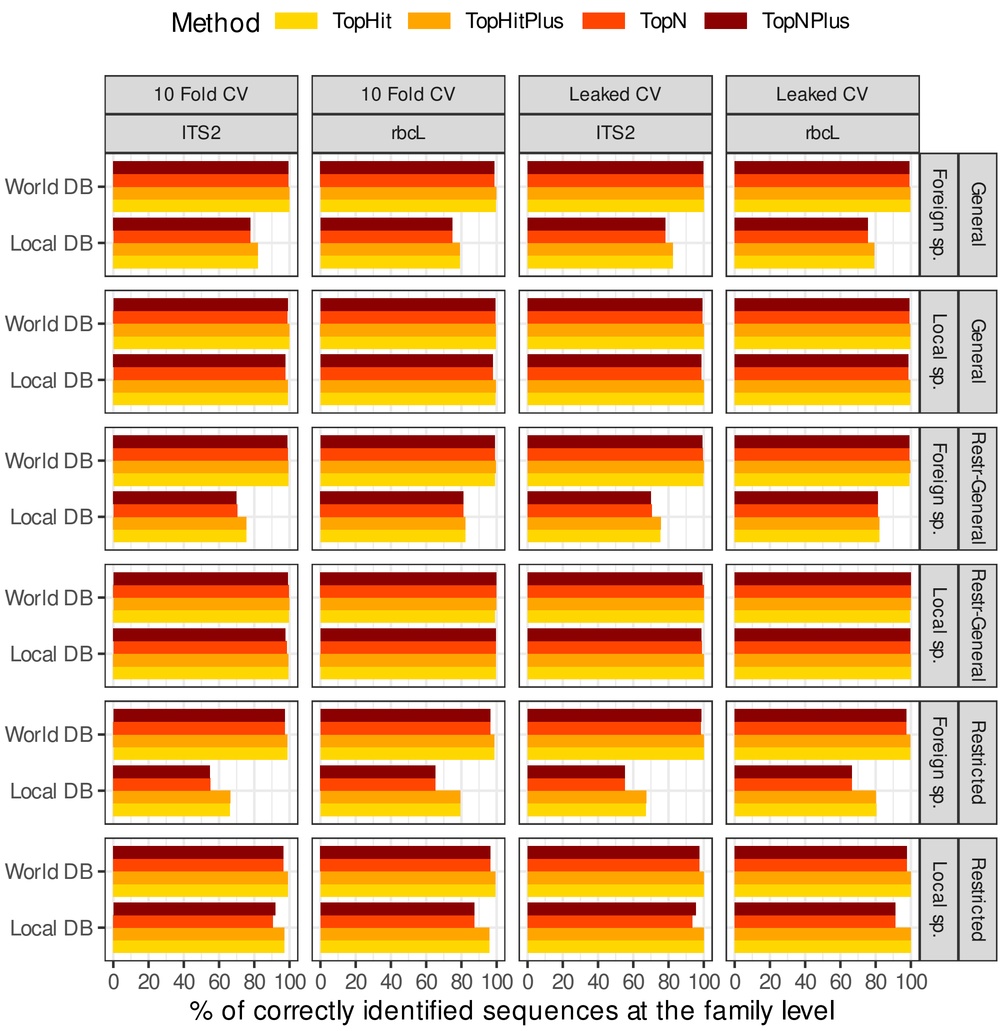

Figure 7:

3.1.5 % correct predictions at the order level

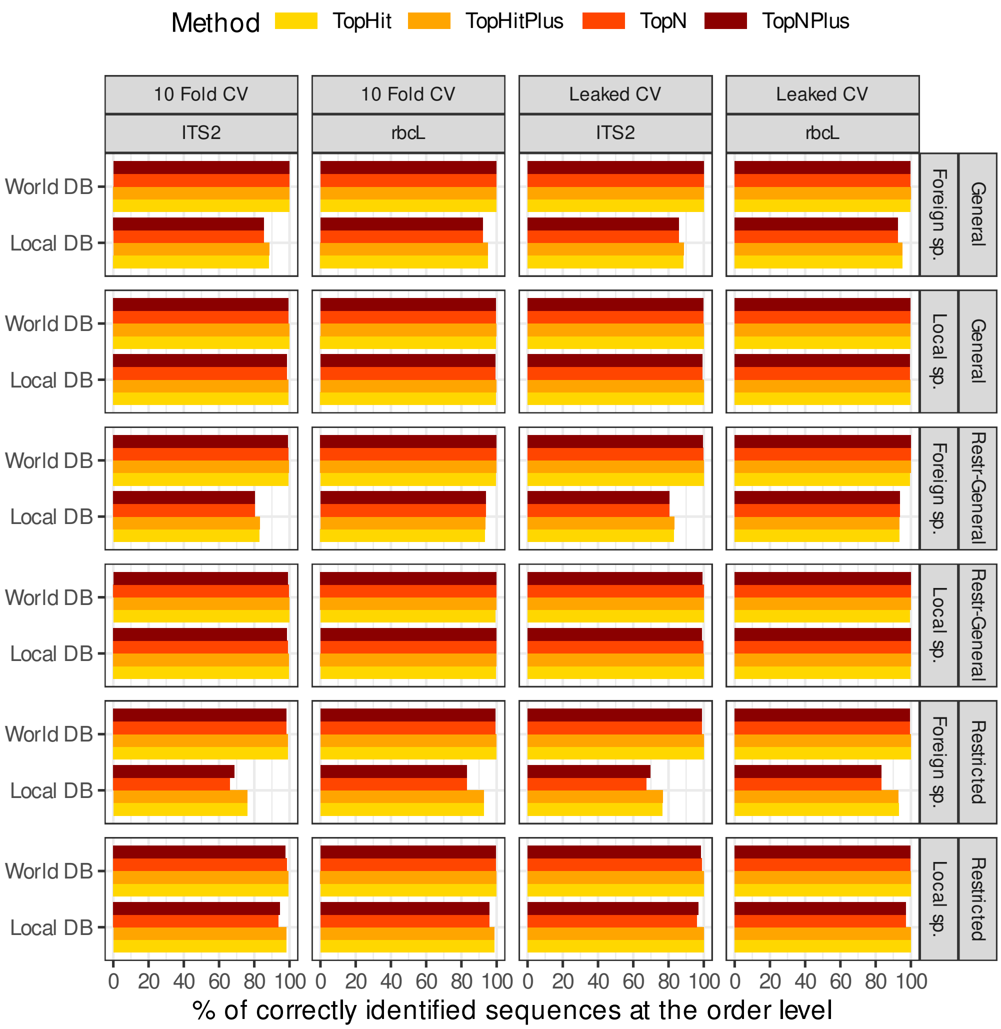

Figure 8:

3.2 Comparisons

3.2.1 Compare the 4 taxonomic assignation methods

Compare the 4 taxonomic assignation methods : TopN (top 10 here, based on consensus score) methods never outperform TopHit methods (= Top 1 based on bit score).

TopHitPlus method slightly outperforms TopHit method in several circumstances, particularly for rbcL (most likely because ties are more frequent for rbcL).

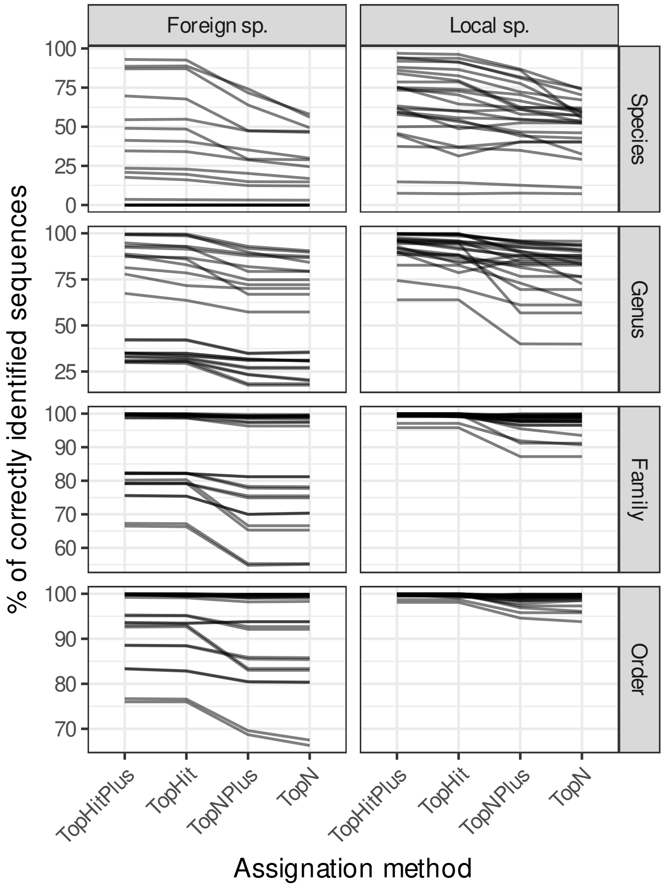

Figure 9:

Same graph but showing the differences relative to TopHitPlus (difference of % of sequences correctly identified)

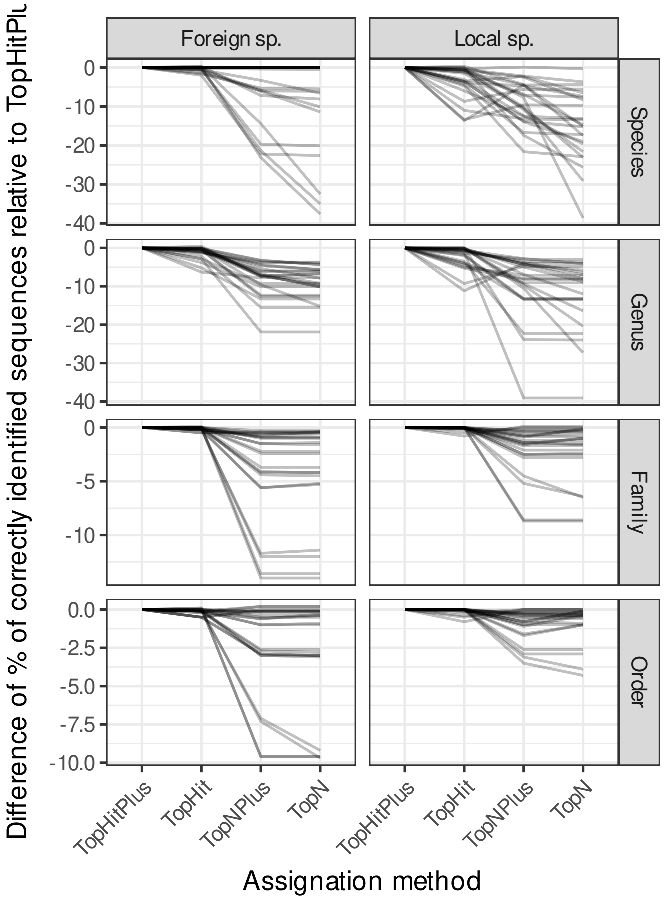

Figure 10:

Table showing the few cases where the other methods than TopHitPlus perform better (with at least > 0.15%).

_

Barcode	CV_method	DB_area	DB_gene	Origin	Tax_level	Method	Diff_pct
ITS2	Leaked CV	Local DB	Restricted	Foreign sp.	Genus	TopHit	0.4
ITS2	10 Fold CV	Local DB	Restricted	Local sp.	Species	TopHit	0.3
rbcL	Leaked CV	World DB	Restricted	Foreign sp.	Species	TopHit	0.3
rbcL	10 Fold CV	Local DB	Restr-General	Foreign sp.	Order	TopN	0.2
rbcL	10 Fold CV	Local DB	Restr-General	Foreign sp.	Order	TopNPlus	0.2
ITS2	Leaked CV	World DB	Restricted	Foreign sp.	Species	TopHit	0.2
rbcL	Leaked CV	Local DB	Restr-General	Foreign sp.	Order	TopN	0.2
rbcL	Leaked CV	Local DB	Restr-General	Foreign sp.	Order	TopNPlus	0.2

Top 20 largest differences between TopHitPlus and TopHit methods (when TopHit performs worse than TopHitPlus). The gain is generally more important for rbcL at both the Species and Genus level

Barcode	CV_method	DB_area	DB_gene	Origin	Tax_level	Method	Diff_pct
rbcL	10 Fold CV	World DB	Restr-General	Local sp.	Species	TopHit	-13.6
rbcL	Leaked CV	World DB	Restr-General	Local sp.	Species	TopHit	-13.4
rbcL	10 Fold CV	World DB	Restr-General	Local sp.	Genus	TopHit	-11.2
rbcL	Leaked CV	World DB	General	Local sp.	Species	TopHit	-11.0
rbcL	Leaked CV	World DB	Restr-General	Local sp.	Genus	TopHit	-9.3
rbcL	10 Fold CV	World DB	General	Local sp.	Species	TopHit	-8.7
rbcL	10 Fold CV	World DB	Restr-General	Foreign sp.	Genus	TopHit	-6.3
rbcL	10 Fold CV	Local DB	Restr-General	Local sp.	Species	TopHit	-6.0
rbcL	10 Fold CV	Local DB	Restr-General	Local sp.	Genus	TopHit	-5.3
rbcL	Leaked CV	World DB	Restr-General	Foreign sp.	Genus	TopHit	-5.2
rbcL	10 Fold CV	World DB	General	Local sp.	Genus	TopHit	-5.0
rbcL	Leaked CV	Local DB	General	Local sp.	Species	TopHit	-4.7
rbcL	Leaked CV	World DB	General	Local sp.	Genus	TopHit	-4.5
rbcL	Leaked CV	Local DB	Restr-General	Local sp.	Species	TopHit	-4.4
rbcL	10 Fold CV	World DB	Restricted	Local sp.	Genus	TopHit	-4.1
ITS2	10 Fold CV	World DB	Restr-General	Local sp.	Species	TopHit	-3.9
rbcL	Leaked CV	Local DB	Restr-General	Local sp.	Genus	TopHit	-3.9
rbcL	10 Fold CV	World DB	Restricted	Foreign sp.	Genus	TopHit	-3.8
rbcL	10 Fold CV	Local DB	General	Local sp.	Species	TopHit	-3.6
ITS2	Leaked CV	World DB	Restr-General	Local sp.	Species	TopHit	-3.6

3.2.2 Compare 10 fold CV vs leaked CV & General vs Restricted

The following graph shows the results for the TopHitPlus assignation method only (which provides the best results).

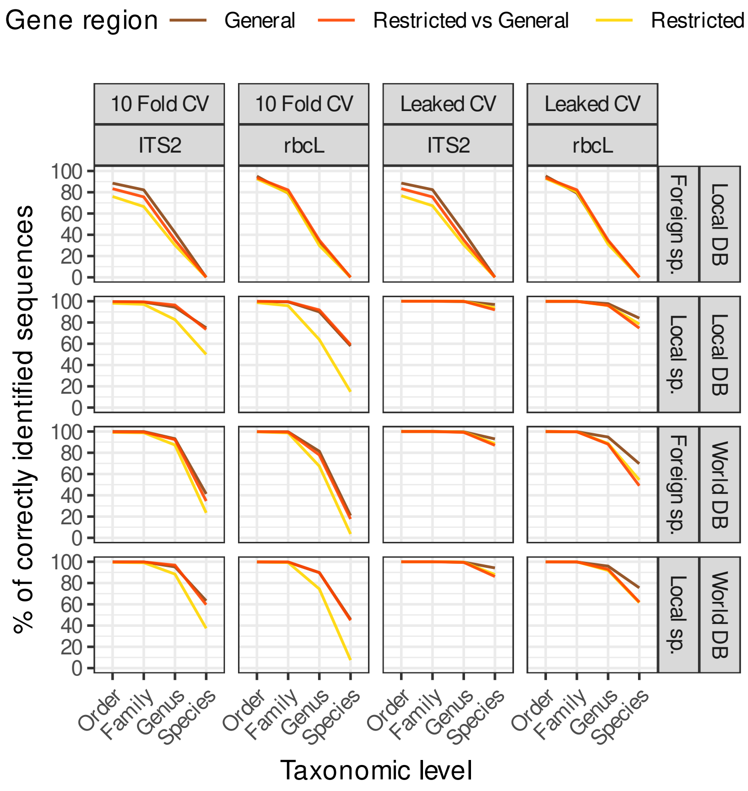

Figure 11:

Leaked CV vs 10 fold CV results :

- With leaked cross validation the % of correctly identified sequences is always better than with 10 fold CV as expected.
- However even in this favorable case where we are certain that the exact "right" sequence is present in the data base, the % of correct identification at species level does not reach 100%. This is particularly true for rbcL because this barcode is less specific and the sequences are more similar accross different taxa.
- In real conditions we will not always have the exact sequence in the database. So the true % of correctly identified sequences is probably between the 10 Fold CV results and the Leaked CV results
- NB1 : For the Foreign species blasted against a local DB we don't really have leaked CV : the target sequences of the foreign species is not in the database –> these can be ignored here
- NB2 : these results might change depending on how the database has been dereplicated. For example with a dereplication in majority mode the leaked CV results will be much closer to 100% because identical sequences pointing to different taxa would be assigned to the most frequent taxa. This would give a wrong impression of high accuracy.

General DB vs Restricted DB

When we look at the 10 fold CV :

- The results are much worse than in leaked CV and drop quickly for more precise taxonomic level : in absence of the true sequence, blast struggles to find a pertinent alternative
- The restricted DB performs always worse because the reference database is much smaller –> less easy to find alternative sequences to match
- General and Restricted vs General have similar profiles : the longer sequences from the general database does not necessarily seem to be big advantage in this situation
- Foreign species performs always worse including when the reference database is a World database probably because these foreign taxa are not as well represented in the reference database that the local species (from Northern Europe)

3.2.3 Compare World vs Local database + ITS2 vs rbcL

World vs Local DB

With the general database :

- Using a local DB we gain ~10% at species level for the local species.
- There is no gain at genus level for the local species
- However for foreign species there is a huge gain when we use the World DB particularly at genus level. The performances at genus level are rather similar for foreign and local species when we use a World DB

–> if we want to work mainly at genus level, it is clearly better to work with the world data base.

If we want to work at species level there is some benefit to work with a local DB if we do not expect to have too many foreign species. However the best accuracy obtained here is ~75% of correct assignations at species level with ITS2 for local species blasted against a local Database (and ~58% for rbcL) which means that we have still to accept 25% of incorrect identifications. . . .

ITS2 vs rbcL

At species level ITS2 tends to be 0-20% better than rbcL and ~5-10% at genus level

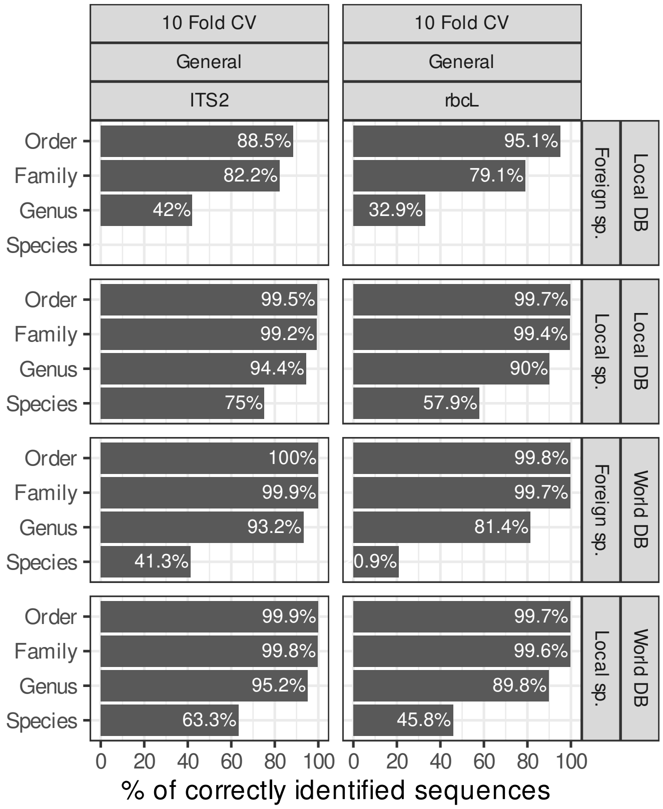

Figure 12:

If we use only the Restricted vs Global data the results are rather similar

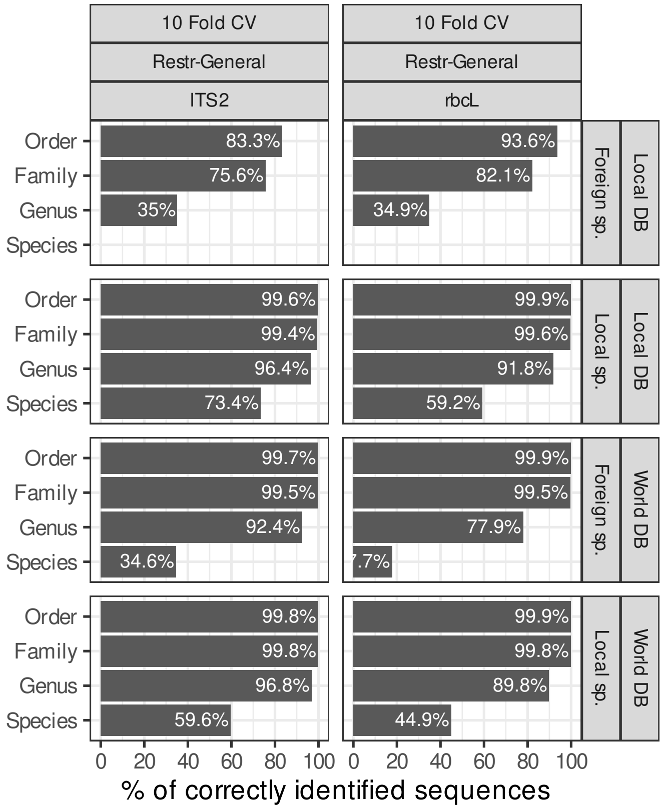

Figure 13:

Finally we can again look in more details at the output of the Leaked CV approach which presents probably over optimistic results. Even in that case, the best results obtained at species level by ITS2 are ~ 95% and for rbcL they vary between ~70 and 84%

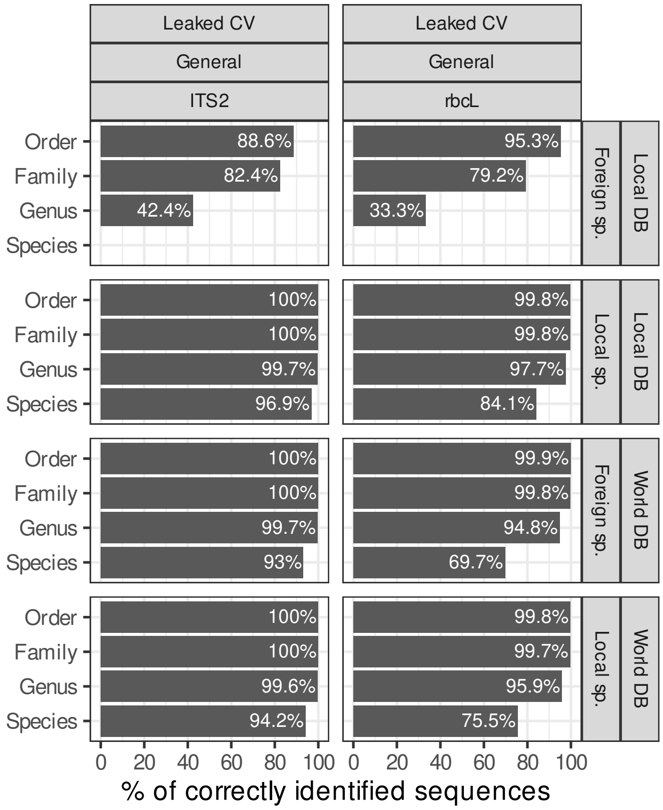

Figure 14:

4 Identity and Consensus scores as predictors of the taxonomic assignation quality

4.1 Simple graphical representation (Single predictor binomial GLMs)

Each blue line is a simple binomial GLM with one predictor (logistic regression). We used the results for `TopHitPlus`, `10 Fold CV`, `General DB` We use a random sample of 10000 sequences to limit graphical overload.

4.1.1 On a World Database

% correct identifications vs Identity score

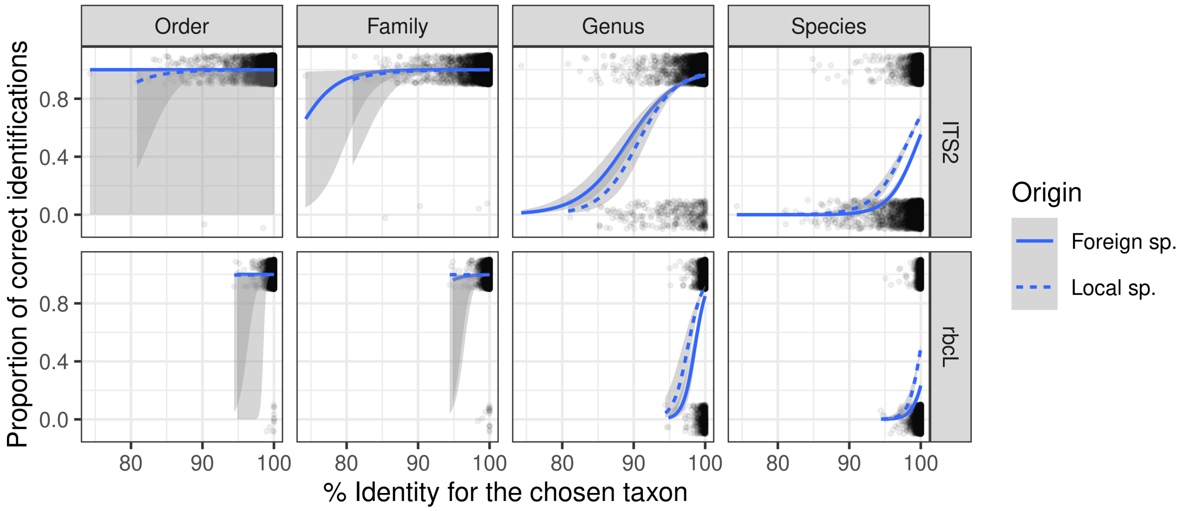

Figure 15:

% correct identification vs Consensus score

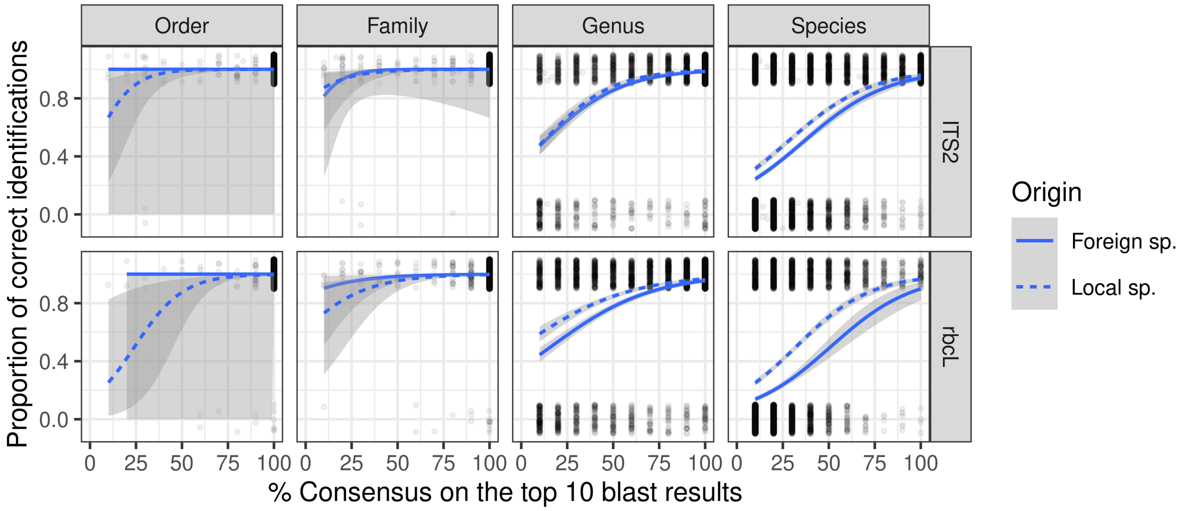

Figure 16:

4.1.2 On a Local Database

(NB : standard errors not displayed because too large for Foreign sp. at species level.)

% correct identifications vs Identity score

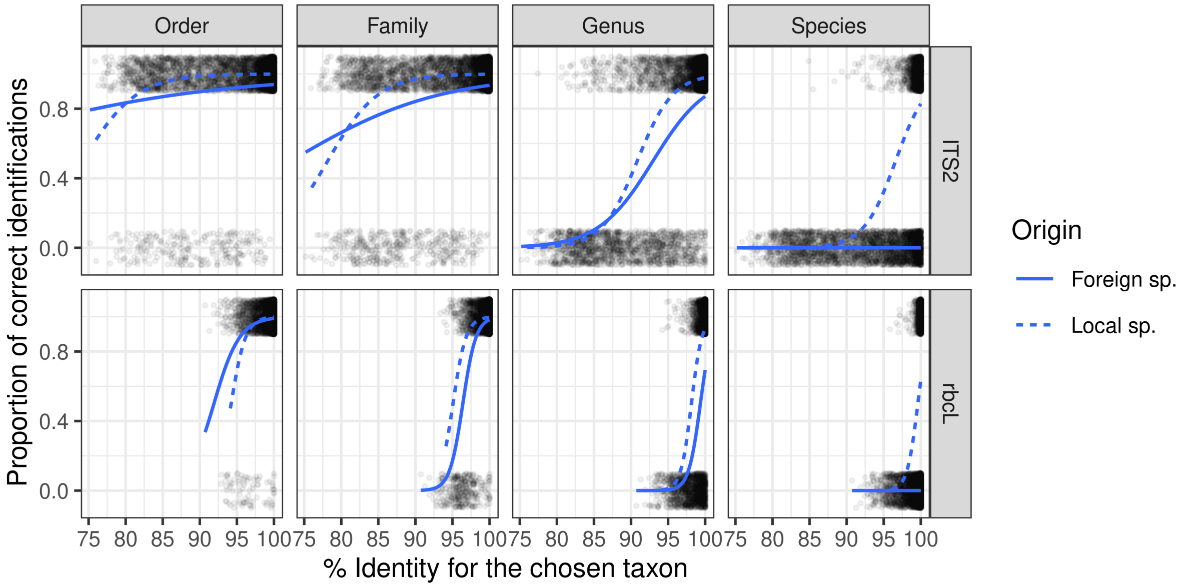

Figure 17:

% correct identification vs Consensus score

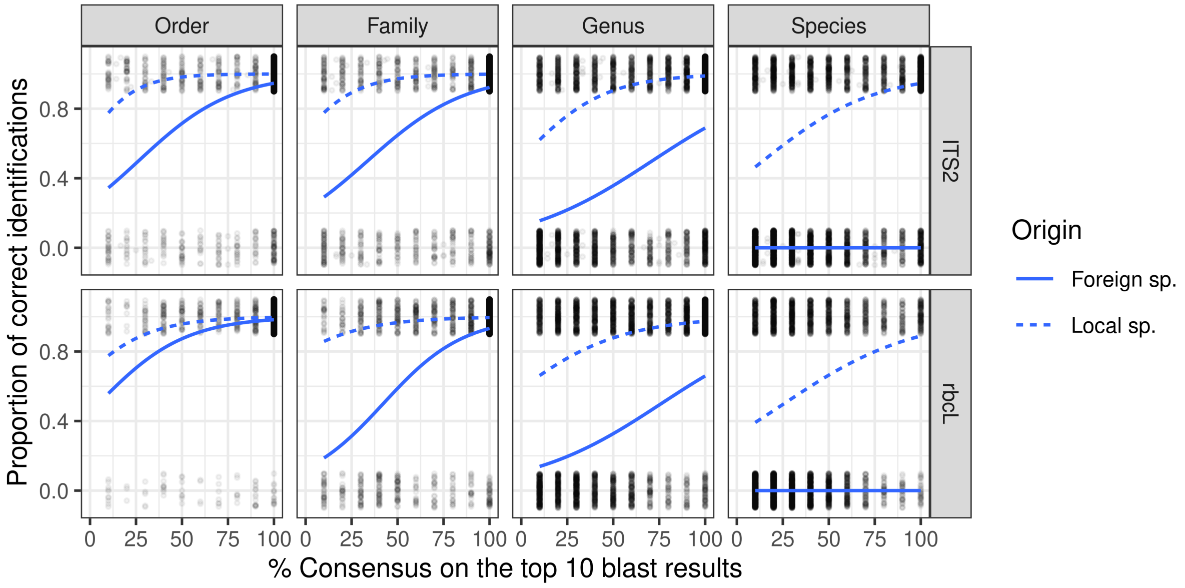

Figure 18:

4.1.3 Distribution of the scores

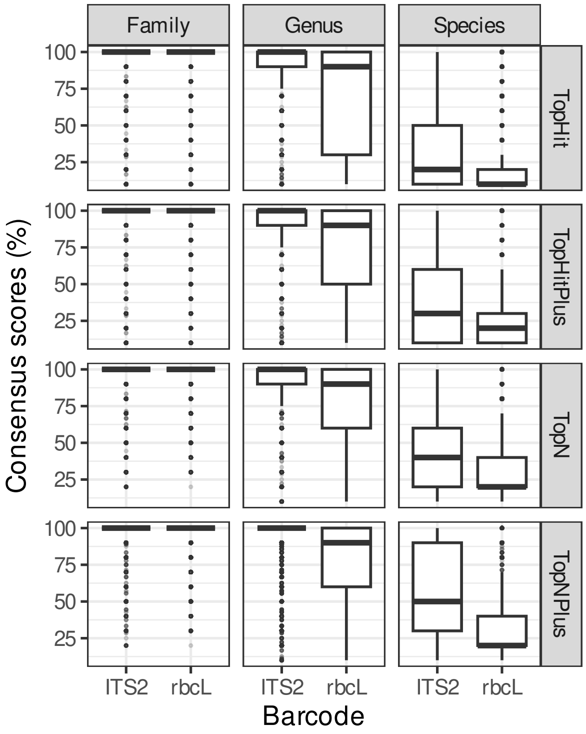

Figure 19:

The 12 graphs on the right are the same as the 12 ones on the left but with a different scale to “zoom in” where most of the values are distributed

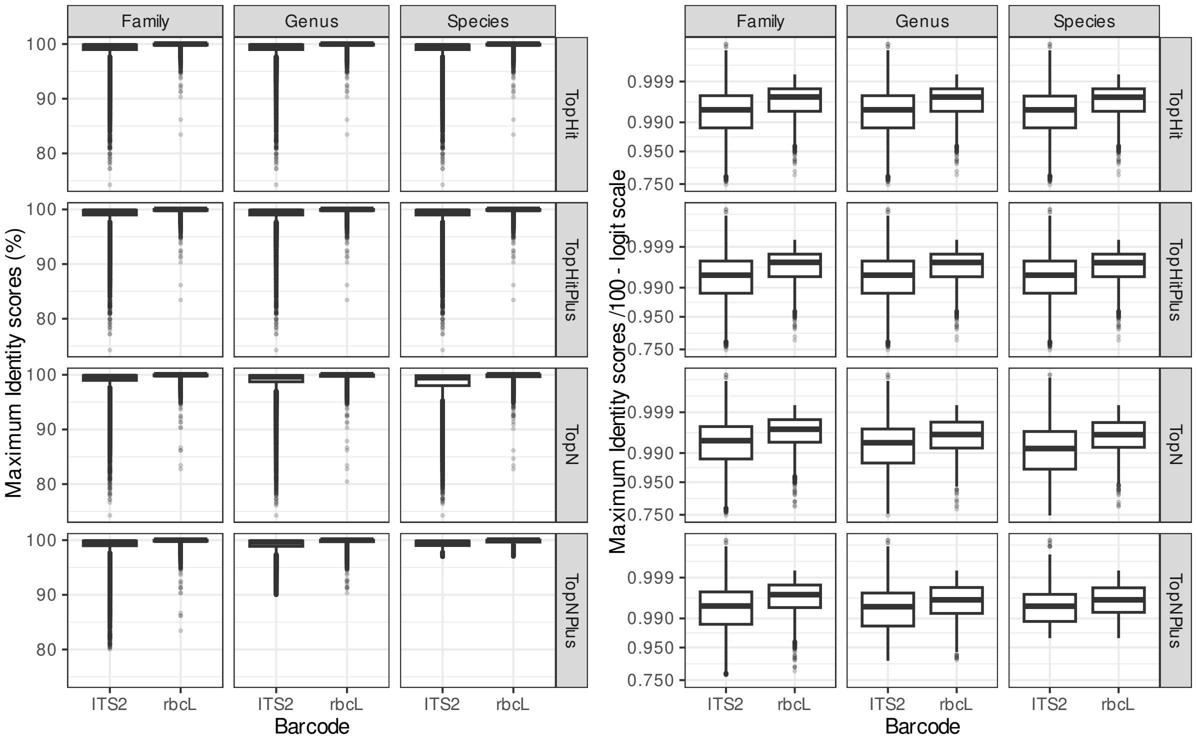
Family
Genus
Species
TopHit
TopHitPlus
TopN
TopNPlus
Maximum Identity scores (%)
100
90
80
ITS2
rbcL
Barcode
Maximum Identity scores /100 - logit scale
0.999
0.990
0.950
0.750

Figure 20:

4.2 Separate multivariate binomial GLMs : `correct ID ~ Consensus * Identity`

We compute a separate binomial GLM `correct ID ~ Consensus * Identity` with a binary response for each combination of strategy and taxonomic level.

4.2.1 Model coefficients

We computed the GLMs only for the 2 Barcodes (ITS2 and rbcL), General and Local DBs and for the Family, Genus and Species taxonomic levels.

In all models the identity score, consensus score and their interactions are significant predictors of the correct/incorrect taxonomic assignation. One exception : ITS2, World DB, Species level : the consensus score and interaction does not seem to be good predictors of the correct/incorrect assignation. However as ssen in the folowing graph there does not seem to have a clear threshold or combination of thresholds that could easily discriminate correct and incorrect taxonomic assignments.

Barcode	DB_area	Tax_level	term	estimate	std.error	statistic	p.value
ITS2	Local DB	Genus	(Intercept)	-26.94590	0.79220	-34.01	0.0000
ITS2	Local DB	Genus	Ident	0.27598	0.00835	33.05	0.0000
ITS2	Local DB	Genus	Cons	-0.07462	0.01133	-6.59	0.0000
ITS2	Local DB	Genus	Ident:Cons	0.00109	0.00012	9.10	0.0000
ITS2	Local DB	Species	(Intercept)	-74.47382	2.21076	-33.69	0.0000
ITS2	Local DB	Species	Ident	0.74621	0.02234	33.40	0.0000
ITS2	Local DB	Species	Cons	0.00462	0.03802	0.12	0.9032
ITS2	Local DB	Species	Ident:Cons	0.00016	0.00038	0.43	0.6681
ITS2	World DB	Genus	(Intercept)	-19.51960	1.47085	-13.27	0.0000
ITS2	World DB	Genus	Ident	0.19768	0.01509	13.10	0.0000
ITS2	World DB	Genus	Cons	-0.05518	0.02169	-2.54	0.0110
ITS2	World DB	Genus	Ident:Cons	0.00101	0.00022	4.55	0.0000
ITS2	World DB	Species	(Intercept)	-44.35024	1.75400	-25.29	0.0000
ITS2	World DB	Species	Ident	0.43449	0.01768	24.57	0.0000
ITS2	World DB	Species	Cons	0.25541	0.03517	7.26	0.0000
ITS2	World DB	Species	Ident:Cons	-0.00215	0.00035	-6.06	0.0000
rbcL	Local DB	Genus	(Intercept)	-178.61613	5.40489	-33.05	0.0000
rbcL	Local DB	Genus	Ident	1.79250	0.05435	32.98	0.0000
rbcL	Local DB	Genus	Cons	0.37032	0.08357	4.43	0.0000
rbcL	Local DB	Genus	Ident:Cons	-0.00345	0.00084	-4.11	0.0000
rbcL	Local DB	Species	(Intercept)	-249.02789	8.93967	-27.86	0.0000
rbcL	Local DB	Species	Ident	2.48499	0.08956	27.75	0.0000
rbcL	Local DB	Species	Cons	0.65649	0.18321	3.58	0.0003
rbcL	Local DB	Species	Ident:Cons	-0.00636	0.00184	-3.47	0.0005
rbcL	World DB	Genus	(Intercept)	-119.64769	8.62119	-13.88	0.0000
rbcL	World DB	Genus	Ident	1.19712	0.08646	13.85	0.0000
rbcL	World DB	Genus	Cons	0.66733	0.13383	4.99	0.0000
rbcL	World DB	Genus	Ident:Cons	-0.00634	0.00134	-4.72	0.0000
rbcL	World DB	Species	(Intercept)	-111.72490	9.21097	-12.13	0.0000
rbcL	World DB	Species	Ident	1.10044	0.09224	11.93	0.0000
rbcL	World DB	Species	Cons	1.19232	0.28201	4.23	0.0000
rbcL	World DB	Species	Ident:Cons	-0.01144	0.00282	-4.05	0.0001

4.2.2 Distribution of the GLM predicted probabilities

We represent the probability that the identification is correct predicted by these models.

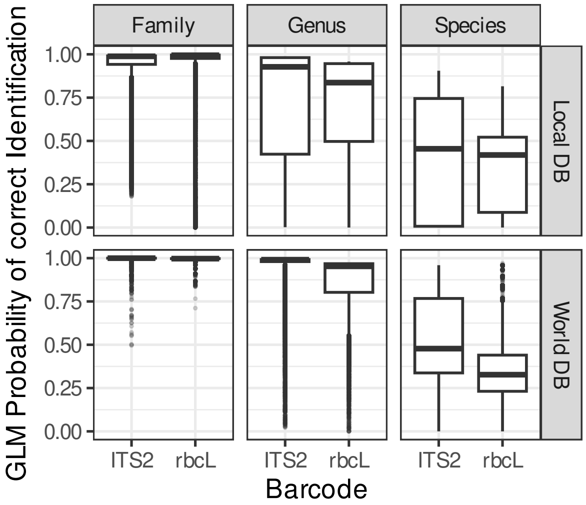

Figure 21:

AUC and pseudo R squared for Both barcodes, Local vs World DB and 3 taxonomic levels :

The pseudo R^2 (`Rsq`) represent the % of variance explained by the model (on a binary response). `Pct` is the % of sequences correctly predicted.

NB these values are not estimated via cross-validation (estimates based on the training set) however with a GLM with only 4 parameters and between 20000 to 30000 sample size there is no overfitting problem and these numbers are totally valid.

The AUC values are generally very good (> 0.9) which means that the identity and Consensus scores combined are generally good to predict wether a taxonomic assignation is correct or no. The AUC tend to be slightly less good for the species level with world databases (~0.8 fopr ITS2 and ~0.75 for rbcL) but these are still informative models.

Barcode	DB_area	Tax_level	AUC	Rsq	Pct
ITS2	Local DB	Family	0.918	0.276	92.1
ITS2	Local DB	Genus	0.946	0.593	70.8
ITS2	Local DB	Species	0.915	0.526	40.8
ITS2	World DB	Family	0.928	0.086	99.9
ITS2	World DB	Genus	0.917	0.306	94.3
ITS2	World DB	Species	0.815	0.295	53.2
rbcL	Local DB	Family	0.962	0.565	91.5
rbcL	Local DB	Genus	0.909	0.510	67.8
rbcL	Local DB	Species	0.816	0.283	35.4
rbcL	World DB	Family	0.766	0.009	99.6
rbcL	World DB	Genus	0.834	0.205	86.6
rbcL	World DB	Species	0.741	0.185	36.1

4.2.3 Graphical representation of predicted probability of correct ID

We show only the results for the General DB, TopHitPlus, 10 Fold CV, World DB

The Points represent the sequences correctly predicted

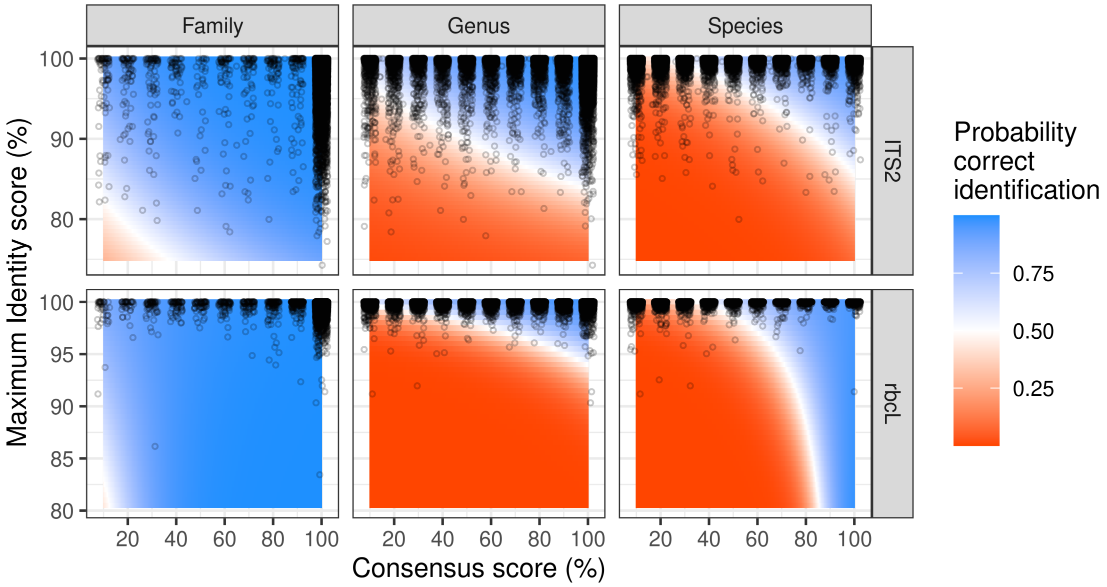

Figure 22:

The Points represent the sequences NOT predicted correctly

The background is exactly identical to the previous graph. We just could not represent both the correct IDs and wrong IDs on the same graph without heavy overload.

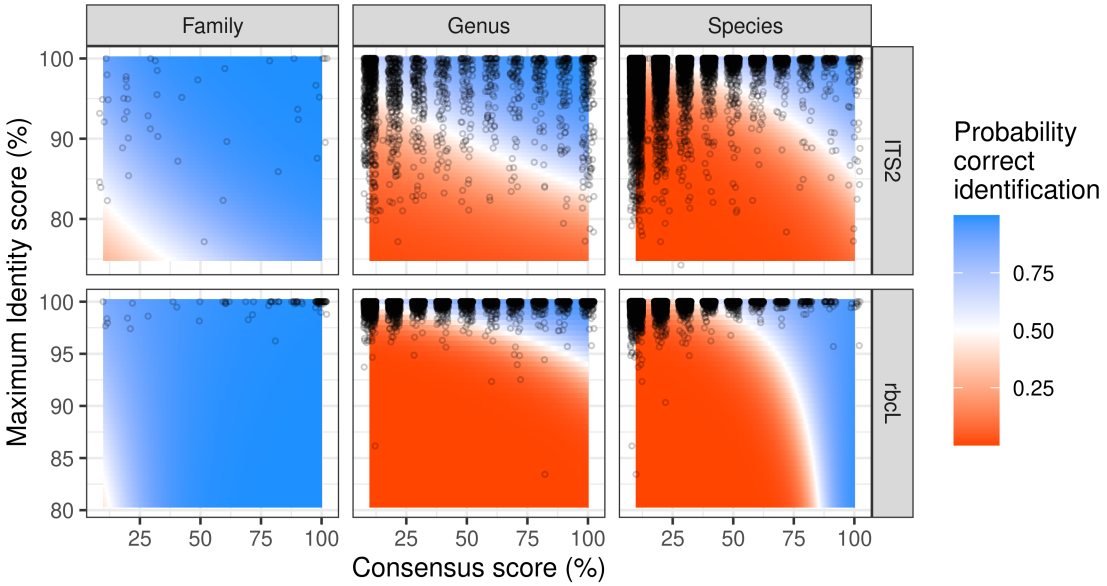

Figure 23:

4.2.4 Using classification trees to help choose a threshold

Prob is the probability of correct ID predicted by the GLMs. We show only the results for the World DB outputs.

rbcL

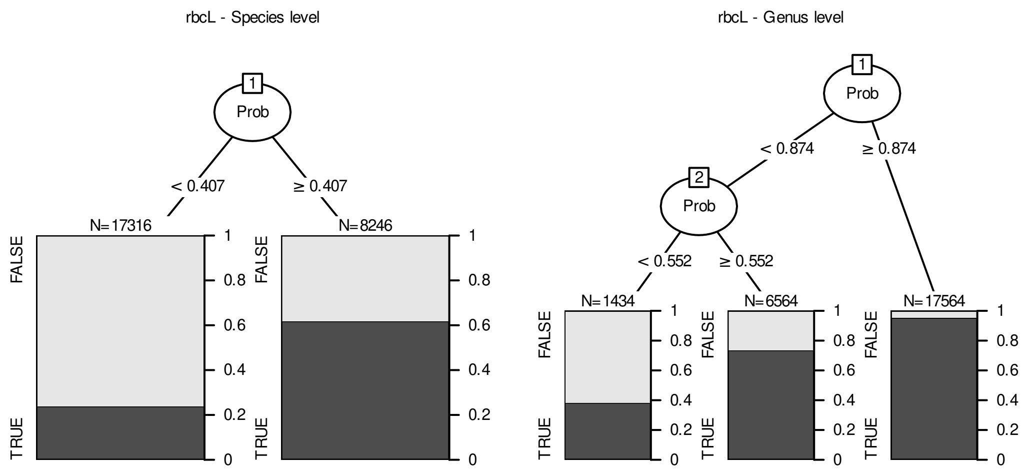

Figure 24:

ITS2

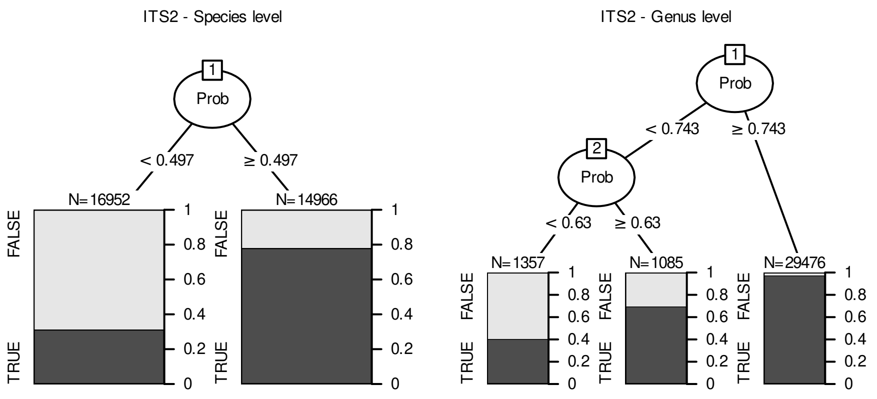

Figure 25:

4.3 Classification trees : predict if the ID is correct at each taxonomic level

Response = binary variable : Correct identification (TRUE or FALSE). Two predictors : Consensus and Identity score. Separate models for each barcode, taxonomic level and World vs Local database (for the genus level we fitted models only for the World DB because it provides always the best results). Trees pruned with 1 standard error rule on the cross-validation error.

4.3.1 Species level - ITS2 - Local DB

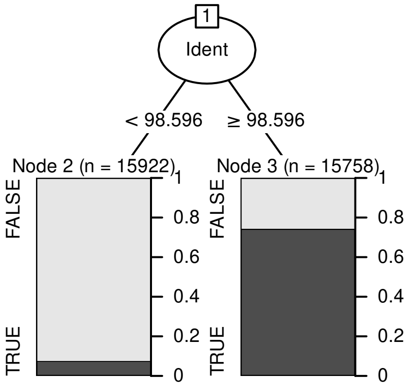

Figure 26:

4.3.2 Species level - rbcL - Local DB

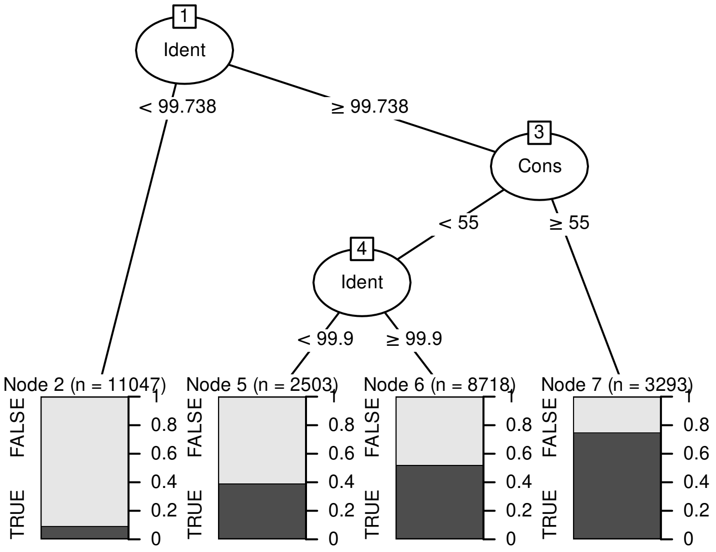

Figure 27:

4.3.3 Species level - ITS2 - World DB

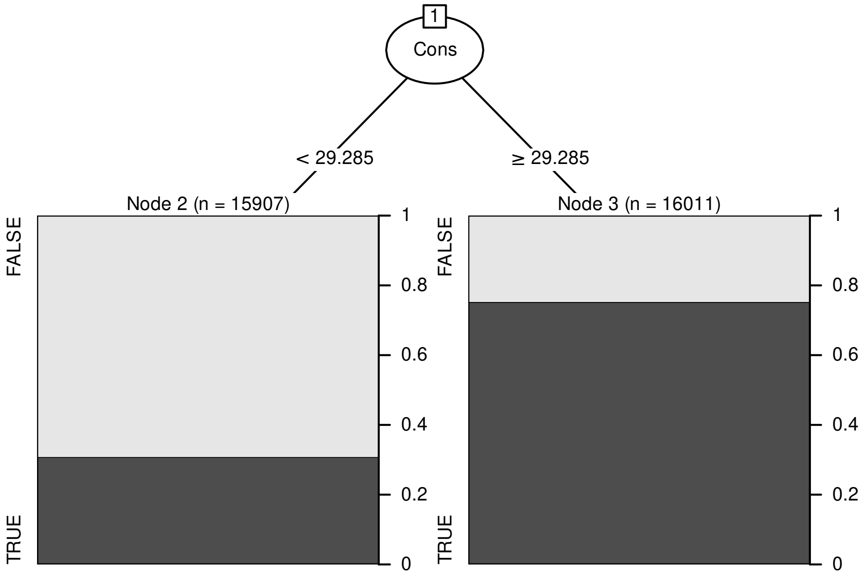

Figure 28:

4.3.4 Species level - rbcL - World DB

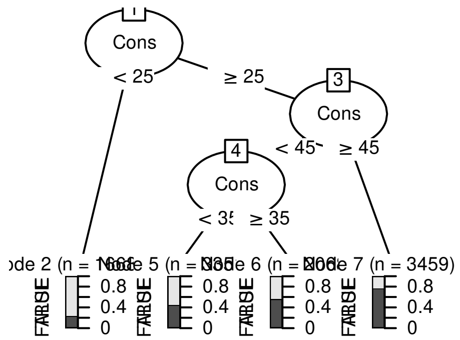

Figure 29:

4.3.5 Genus level - ITS2

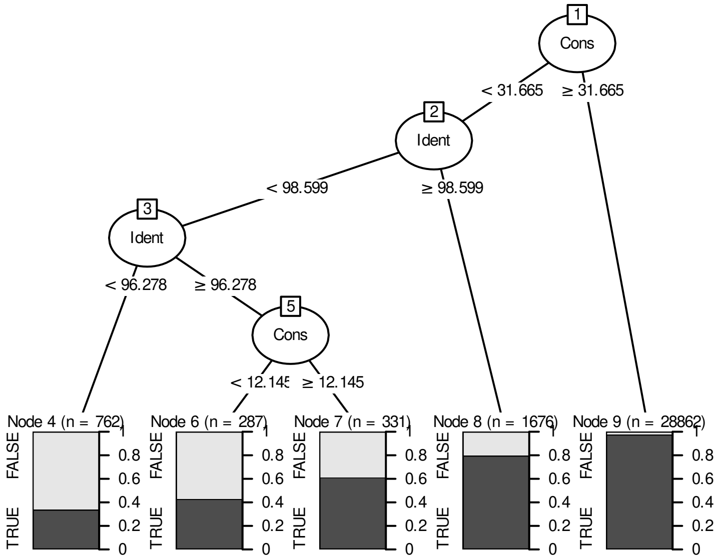

Figure 30:

4.3.6 Genus level - rbcL

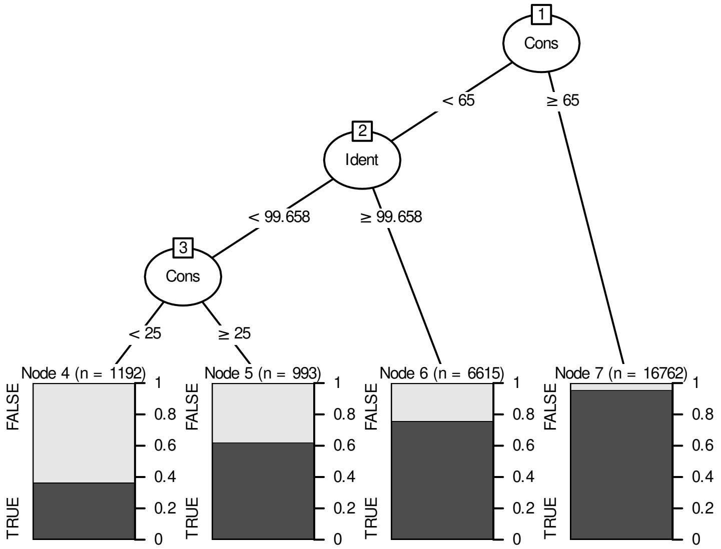

Figure 31:

4.4 Classification trees : Predict the best taxonomic level with identity and consensus scores

The approach here is slightly different. Instead of predicting if the identification is correct and model this response at each taxonomic level, the response is here the best taxonomic level with correct identification. So the classification tree corresponds to the folloing model :

```
factor(TaxScore) ~ s_Ident + s_Cons + g_Ident + g_Cons + f_Ident + f_Cons
```

Where `TaxScore` is the most precise correct taxonomic level : 7 = species, 6 = genus, 5 = family, 4 = order, 3 = not correct even at the Order level –> unknown taxon.

4.4.1 ITS2

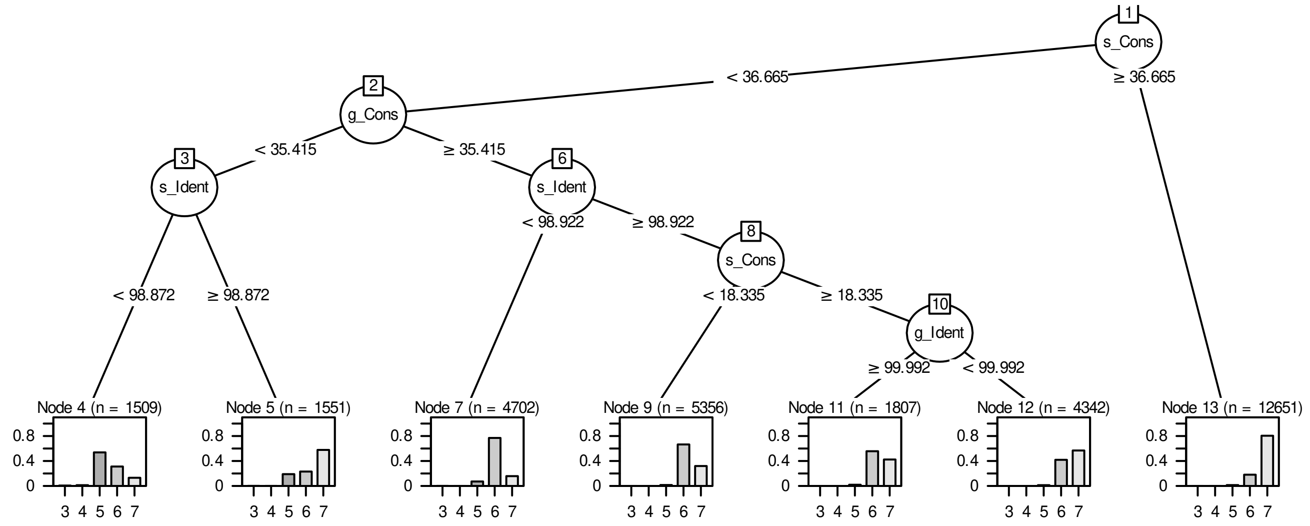

Figure 32:

4.4.2 rbcL

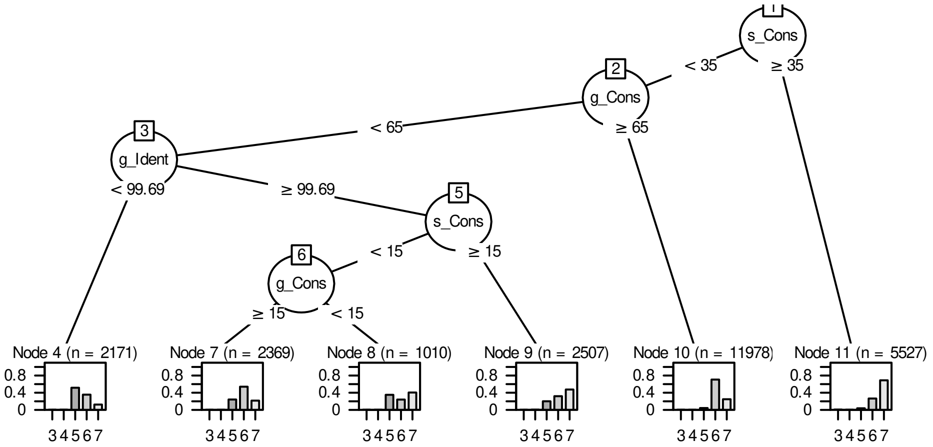

Figure 33:

4.5 Reduction of prediction error when we apply exclusion rules based on the Classification trees

At the genus level, we use only the World DB results and discard the taxonomic assignations with Consensus < 40% and Identity < 98.5% for ITS2 or Identity < 99.7% for rbcL

At the species level, we first check the Local DB prediction, then if it does not fulfill the quality criteria, we use the World DB prediction if this one fulfills other quality criteria. If none of the DB provides a satisfying prediction, no taxon is assigned at the species level ("Unknown identification").

For ITS2, with a Local DB, we discard all predictions with Identity < 99%. Then we try the World DB and discard all sequences with a Consensus < 40% and an Identity < 99.9% and we also discard all sequences with a consensus < 20% (even if their Identity is > 99.9)

For rbcL, with a Local DB, we discard all predictions with Identity < 99.7% or with an Identity <= 100% and a Consensus < 50%. Then we try the World DB and discard all sequences with a Consensus < 50%

On the following graph we compare the results for the raw assignation (without filtering out the untrustworthy predictions - based on the World DB) and for the assignations after eliminating the sequences which do not fulfill these criteria.

If the rules worked perfectly, all the wrong identification from the raw assignments should be transferred to "unknown identification" and none of the raw correct identifications should be transferred to the Unknown identification category…

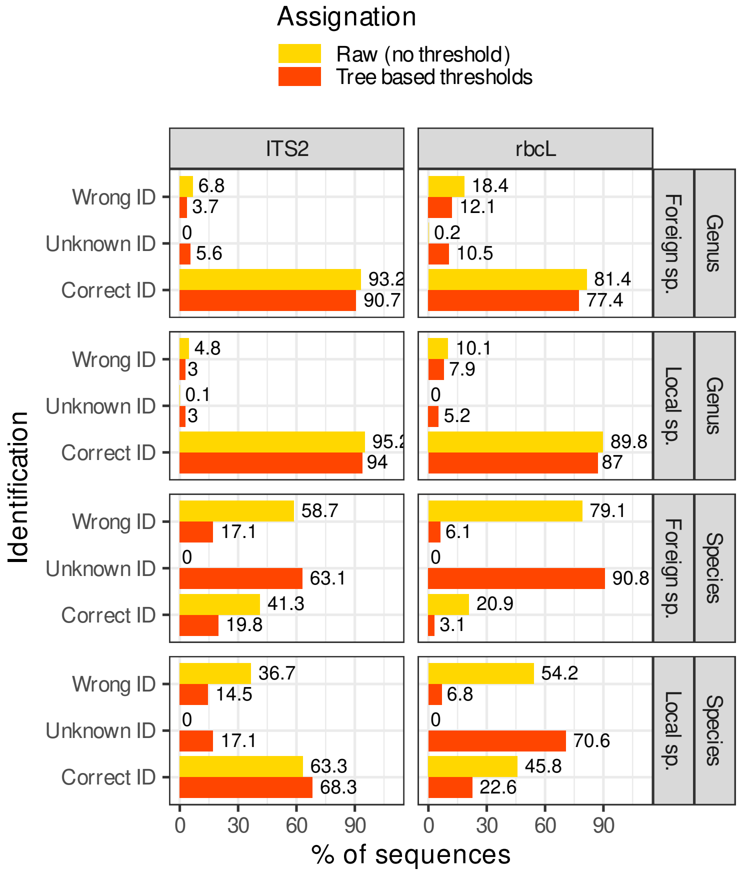

Figure 34:

Same approach but with Local and Foreign species grouped

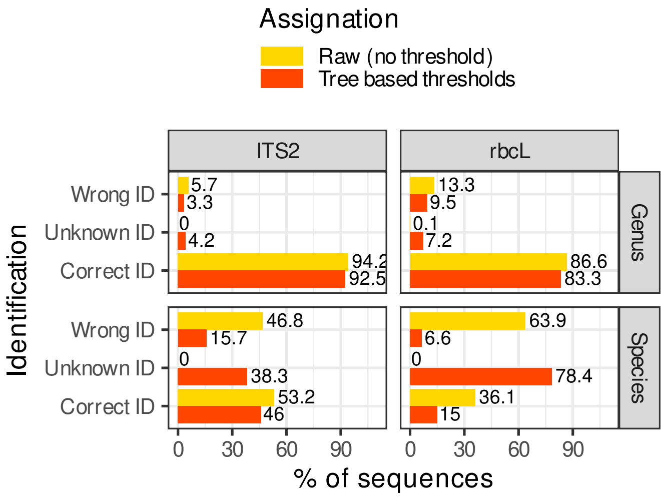

Figure 35:

5 Compare ITS2 and rbcL for various taxonomic groups

In this section we want to explore whether maybe some plant families are better identified than others or whether certain are better identified with ITS2 or rbcL.

5.1 % of correctly identified sequences for each Family

When we don't take all sequences into account, there are several classical ways to compute the % of correctly identified sequences :

- P = Precision = % of Taxon_predicted which is correct `P = TP/(TP+FP)`
- R = Recall = % of Taxon_true for which the prediction is correct `R = TP/(TP+FN)`
- F = `F-score` = harmonic mean of F and P `F = 2*P*R/(P+R)`

The problem is that there are in fact several ways to combine these values to obtain a global estimate. For example, our question of interest could be : for all sequences that are truly in the family "Rosaceae", which proportion are correctly predicted at family level (this is = to the Recall, but of limited interest here) but also at the genus and species level ?

One possibility is to compute F, R and P for each species and genus and then compute their average for each family to obtain a global estimate of the the prediction quality at at the genus and species level for each family. The difficulty with this approach is that there are lots of NAs and 0 (for example if a genus is never assigned, its Precision will be NA) and it is difficult to choose how to combine them. This typically occurs in species poorly represented in the database and can have a huge influence on the results. We will not use this approach here but we can still examine the R, P and F values for some genus of interest.

Another possibility is simply to compute the % of correct prediction across all sequences for each true family at genus and species level. With this approach the species or genus with a higher number of sequences will have a higher weight on the final result. See the examples in the help of the function `score_per_taxon()` from `CVrefDB` package for a demonstration of the similarities and differences between these two approaches.

We present the results for a selection of families that have a lot of sequences and/or are important resources for bees. For example, Poaceae is the family with the highest number of sequences but it is not shown because its importance is lower for bees.

5.1.1 Local species - Local database

Results for 10 Fold CV, General DB, TopHitPlus, Local DB and only for Local species

The following table shows the number of sequences of Local sp. tested (and available in the databases because we tested all sequences corresponding to Local species) in the Local Database for bot ITS2 and rbcL. Hydrophyllaceae, Grossulariaceae, Balsaminaceae, Rhamnaceae and Lythraceae are poorly represented particularly in the ITS2 database (ITS2 : 9-15 sequences, rbcL : 7-30 sequences).

Family	ITS2	rbcL
Asteraceae	1847	1400
Brassicaceae	1251	648
Fabaceae	1025	917
Rosaceae	857	871
Fagaceae	499	78
Sapindaceae	489	162
Caryophyllaceae	416	382
Lamiaceae	399	593
Apiaceae	369	288
Solanaceae	341	359
Orchidaceae	221	189
Ericaceae	196	102
Ranunculaceae	165	259
Campanulaceae	128	192
Convolvulaceae	115	153
Malvaceae	115	123
Boraginaceae	94	136
Hypericaceae	93	52
Pinaceae	81	170
Geraniaceae	73	122
Salicaceae	69	214
Scrophulariaceae	67	64
Balsaminaceae	15	30
Grossulariaceae	15	20
Lythraceae	14	29
Rhamnaceae	12	27
Hydrophyllaceae	9	7

Local species on a Local database :

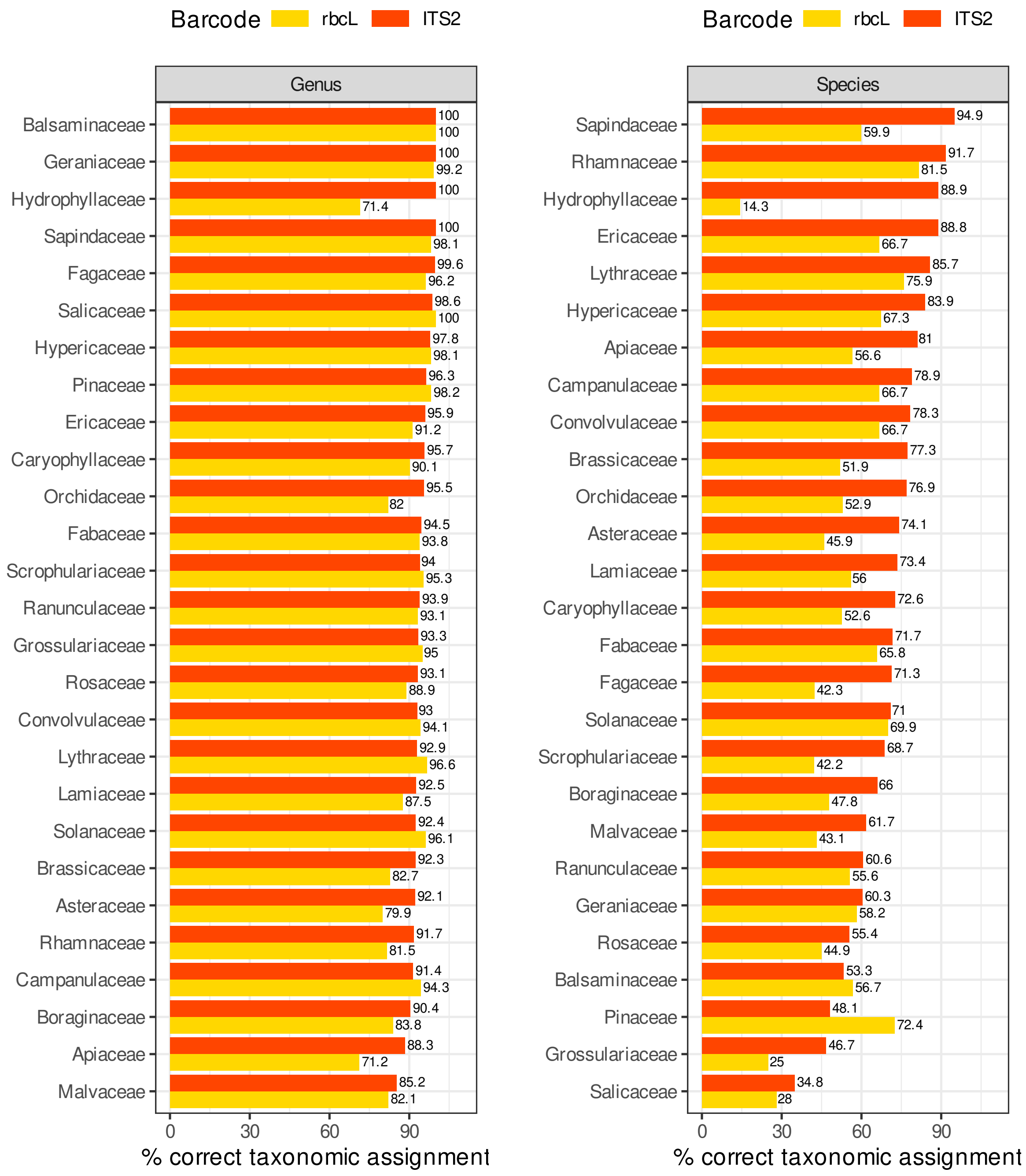

Figure 36:

5.1.2 Local species - World database

Results for 10 Fold CV, General DB, TopHitPlus, World DB and only for Local species

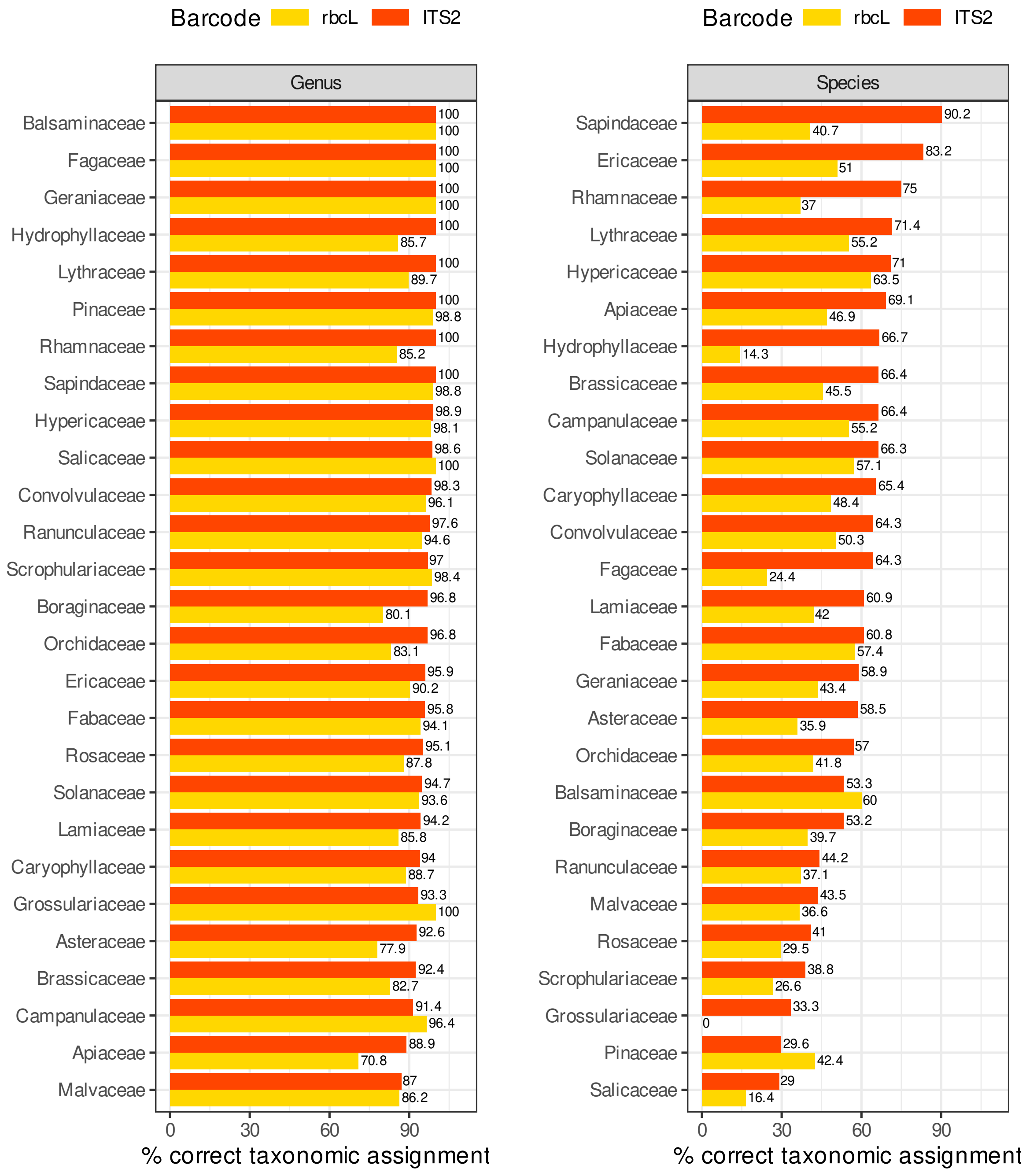

Figure 37:

5.1.3 All species - World database

Results for 10 Fold CV, General DB, TopHitPlus, World DB . We pooled Local and Foreign species.

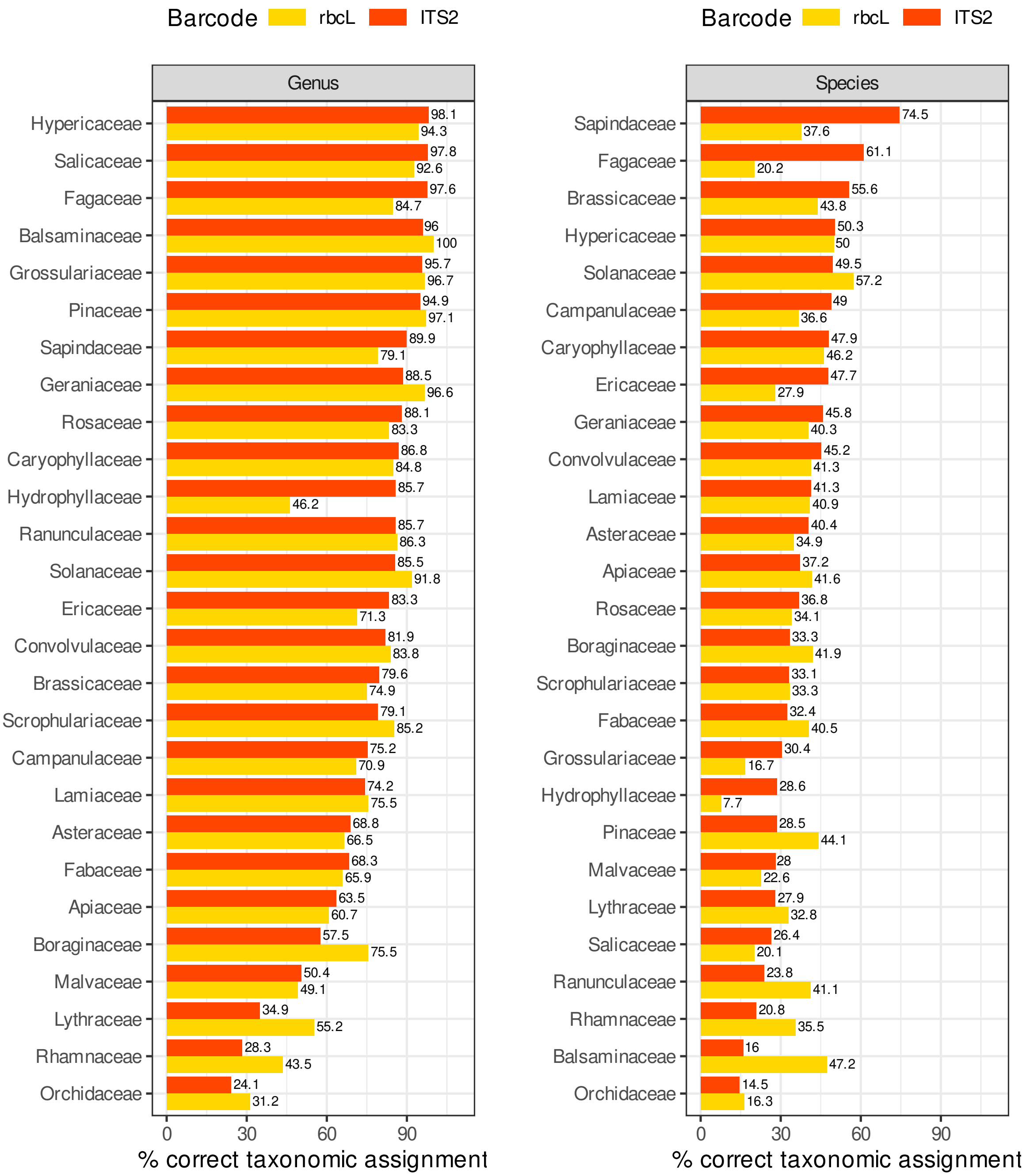

Figure 38:

The following table shows the number of sequences of Local sp. + Foreign species tested against the World Database for bot ITS2 and rbcL. Hydrophyllaceae, Grossulariaceae, Balsaminaceae, Rhamnaceae and Lythraceae are still the less well represented species but the number of seqeunces is higher than when we test only Local species

Quitting from lines 2778-2782 [unnamed-chunk-91] (CV_refDB_02_Data_analysis_Supplements.spin.Rmd) Error in `pivot_wider()`: ! Can't subset columns that don't exist. x Column `Tot` doesn't exist. Backtrace: 1. ... %>% pander 5. tidyr:::pivot_wider.data.frame(., id_cols = Family, names_from = Barcode, values_from = Tot)

5.2 % of correctly identified sequences for various genus of interest

We are particularly interested by "fruit trees" (arborescent Rosaceae) and Brassicaceae because being able to distinguish the genus or species could allow to distinguish between wild sources of pollen or pollen from crops (eg to evaluate the risk of pesticide contamination).

NB : this section is not discussed in the paper.

5.2.1 Fruit trees

How good are the genus level ID of "fruit trees" ?

`Nb` is the number of sequences from the database that have been correctly identified for that genus, `Tot` is the total number of sequences.

We kept only the sequences for which the true genus or the predicted genus is among the main "fruit trees" : "Malus", "Pyrus", "Prunus", "Crataegus", "Sorbus"

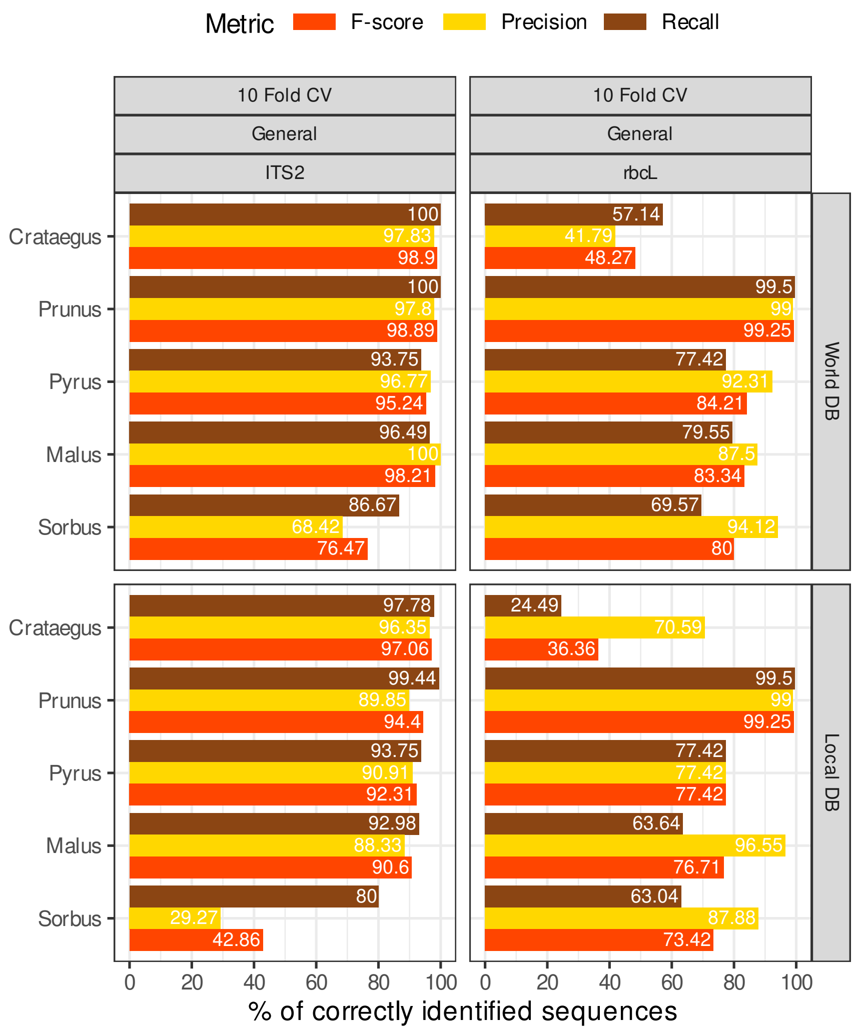

Figure 39:

The following table shows all the cases where the genus was wrongly identified and for which either the target genus or the predicted genus is the genus "Sorbus"

Only results for 10 Fold CV, General, World DB and TopHitPlus. The Local and foreign species are pooled.

TaxID_query	Barcode	Method	Species_true	Wild	Genus	g_Ident	g_Cons
U16188.1	ITS2	TopHitPlus	Cotoneaster coriaceus	Introduced	Sorbus	89.809	10
MN577923.1	ITS2	TopHitPlus	Gillenia trifoliata	Absent	Sorbus	96.808	20
DQ811764.1	ITS2	TopHitPlus	Kageneckia angustifolia	Absent	Sorbus	87.284	10
U16195.1	ITS2	TopHitPlus	Malus domestica	Introduced	Sorbus	90.948	10
U16204.1	ITS2	TopHitPlus	Sorbus aucuparia	Wild	Malacomeles	93.926	10
AF186533.1	ITS2	TopHitPlus	Sorbus torminalis	Wild	Torminalis	97.293	10
MN215989.1	ITS2	TopHitPlus	Stranvaesia nussia	Absent	Sorbus	98.082	40
MN215999.1	ITS2	TopHitPlus	Torminalis clusii	Absent	Sorbus	98.627	60
KT992459.1	rbcL	TopHitPlus	Amelanchier alnifolia	Absent	Sorbus	99.672	30
HG765052.1	rbcL	TopHitPlus	Crataegus monogyna	Wild	Sorbus	100.000	30
DQ860482.1	rbcL	TopHitPlus	Sorbus alnifolia	Absent	Aronia	100.000	10
DQ860504.1	rbcL	TopHitPlus	Sorbus americana	Absent	Aronia	100.000	10
HG765087.1	rbcL	TopHitPlus	Sorbus aria	Wild	Amelanchier	99.859	50
HG765088.1	rbcL	TopHitPlus	Sorbus aucuparia	Wild	Amelanchier	100.000	60
MG703646.1	rbcL	TopHitPlus	Sorbus aucuparia	Wild	Micromeles	100.000	10
HE963687.1	rbcL	TopHitPlus	Sorbus domestica	Introduced	Amelanchier	100.000	40
HG765090.1	rbcL	TopHitPlus	Sorbus domestica	Introduced	Amelanchier	100.000	60
JN890680.1	rbcL	TopHitPlus	Sorbus domestica	Introduced	Amelanchier	100.000	40
JN891096.1	rbcL	TopHitPlus	Sorbus domestica	Introduced	Amelanchier	100.000	40
KM360990.1	rbcL	TopHitPlus	Sorbus domestica	Introduced	Amelanchier	99.645	100
MH116420.1	rbcL	TopHitPlus	Sorbus hupehensis	Absent	Amelanchier	100.000	70
KX371925.1	rbcL	TopHitPlus	Sorbus scopulina	Absent	Amelanchier	100.000	40
KY457242.1	rbcL	TopHitPlus	Sorbus torminalis	Wild	Torminalis	100.000	10
MH657683.1	rbcL	TopHitPlus	Sorbus tsinlingensis	Absent	Amelanchier	100.000	30

Idem for Pyrus

TaxID_query	Barcode	Method	Species_true	Wild	Genus	g_Ident	g_Cons
MG237692.1	ITS2	TopHitPlus	Chaenomeles japonica	Introduced	Pyrus	95.070	70
U16202.1	ITS2	TopHitPlus	Pyrus calleryana	Introduced	Chaenomeles	90.812	10
MN577903.1	ITS2	TopHitPlus	Pyrus communis	Introduced	Photinia	98.057	10
AB603907.1	rbcL	TopHitPlus	Malus domestica	Introduced	Pyrus	98.387	100
MK920295.1	rbcL	TopHitPlus	Mespilus germanica	Wild	Pyrus	99.930	60
AB603918.1	rbcL	TopHitPlus	Pyrus communis	Introduced	Rhaphiolepis	100.000	50
DQ860502.1	rbcL	TopHitPlus	Pyrus communis	Introduced	Aronia	100.000	10
JN892983.1	rbcL	TopHitPlus	Pyrus communis	Introduced	Cotoneaster	100.000	60
JN893266.1	rbcL	TopHitPlus	Pyrus communis	Introduced	Cotoneaster	100.000	60
JQ391213.1	rbcL	TopHitPlus	Pyrus communis	Introduced	Crataegus	100.000	80
JQ391209.1	rbcL	TopHitPlus	Pyrus salicifolia	Introduced	Crataegus	100.000	80
AB603911.1	rbcL	TopHitPlus	Pyrus ussuriensis	Absent	Rhaphiolepis	100.000	50

Idem for Malus

TaxID_query	Barcode	Method	Species_true	Wild	Genus	g_Ident	g_Cons
GQ436116.1	ITS2	TopHitPlus	Malus baccata	Introduced	Ziziphus	97.741	20
U16195.1	ITS2	TopHitPlus	Malus domestica	Introduced	Sorbus	90.948	10
MN473033.1	rbcL	TopHitPlus	Cercocarpus rzedowskii	Absent	Malus	100.000	60
MN068267.1	rbcL	TopHitPlus	Malacomeles denticulata	Absent	Malus	99.860	30
JQ391176.1	rbcL	TopHitPlus	Malus baccata	Introduced	Crataegus	100.000	80
AB603907.1	rbcL	TopHitPlus	Malus domestica	Introduced	Pyrus	98.387	100
JQ391187.1	rbcL	TopHitPlus	Malus ioensis	Absent	Crataegus	100.000	100
JQ391175.1	rbcL	TopHitPlus	Malus mandshurica	Absent	Crataegus	100.000	80
JQ391185.1	rbcL	TopHitPlus	Malus sieboldii	Introduced	Crataegus	100.000	90
JQ391200.1	rbcL	TopHitPlus	Malus sieversii	Absent	Crataegus	100.000	80
JQ391194.1	rbcL	TopHitPlus	Malus sylvestris	Wild	Crataegus	100.000	80
KX499858.1	rbcL	TopHitPlus	Malus trilobata	Absent	Amelanchier	99.860	40
JQ391172.1	rbcL	TopHitPlus	Malus zhaojiaoensis	Absent	Crataegus	100.000	80

TaxID_query	Barcode	Method	Species_true	Wild	Genus	g_Ident	g_Cons
KC251298.1	rbcL	TopHitPlus	Mespilus germanica	Wild	Malus	100.000	100
KY420011.1	rbcL	TopHitPlus	Peraphyllum ramosissimum	Absent	Malus	99.860	20
LC413406.1	rbcL	TopHitPlus	Rhaphiolepis umbellata	Absent	Malus	100.000	30

Idem for Crataegus

TaxID_query	Barcode	Method	Species_true	Wild	Genus	g_Ident	g_Cons
GQ436114.1	ITS2	TopHitPlus	Chaenomeles speciosa	Introduced	Crataegus	99.589	10
GQ436587.1	ITS2	TopHitPlus	Chaenomeles speciosa	Introduced	Crataegus	99.716	10
MN215986.1	ITS2	TopHitPlus	Mespilus germanica	Wild	Crataegus	98.310	10
HG416962.1	rbcL	TopHitPlus	Amelanchier ovalis	Wild	Crataegus	100.000	50
JQ391078.1	rbcL	TopHitPlus	Amelanchier ovalis	Wild	Crataegus	100.000	80
JQ391083.1	rbcL	TopHitPlus	Amelanchier spicata	Introduced	Crataegus	100.000	90
JQ391092.1	rbcL	TopHitPlus	Aronia melanocarpa	Introduced	Crataegus	100.000	80
JQ391145.1	rbcL	TopHitPlus	Chaenomeles japonica	Introduced	Crataegus	100.000	100
JQ391161.1	rbcL	TopHitPlus	Chaenomeles sinensis	Absent	Crataegus	100.000	100
JQ391142.1	rbcL	TopHitPlus	Chaenomeles speciosa	Introduced	Crataegus	100.000	100
JQ391122.1	rbcL	TopHitPlus	Cotoneaster acuminatus	Absent	Crataegus	100.000	90
JQ391094.1	rbcL	TopHitPlus	Cotoneaster acutifolius	Absent	Crataegus	100.000	80
JQ391104.1	rbcL	TopHitPlus	Cotoneaster ambiguus	Introduced	Crataegus	100.000	80
JQ391096.1	rbcL	TopHitPlus	Cotoneaster assamensis	Absent	Crataegus	100.000	80
JQ391110.1	rbcL	TopHitPlus	Cotoneaster bullatus	Introduced	Crataegus	100.000	80
JQ391126.1	rbcL	TopHitPlus	Cotoneaster dielsianus	Wild	Crataegus	100.000	80
JQ391131.1	rbcL	TopHitPlus	Cotoneaster divaricatus	Wild	Crataegus	100.000	80
JQ391113.1	rbcL	TopHitPlus	Cotoneaster foveolatus	Introduced	Crataegus	100.000	80
JQ391130.1	rbcL	TopHitPlus	Cotoneaster franchetii	Wild	Crataegus	100.000	90
JQ391133.1	rbcL	TopHitPlus	Cotoneaster horizontalis	Wild	Crataegus	100.000	80
JQ391106.1	rbcL	TopHitPlus	Cotoneaster integerrimus	Wild	Crataegus	100.000	80
JQ391095.1	rbcL	TopHitPlus	Cotoneaster moupinensis	Introduced	Crataegus	100.000	80
JQ391129.1	rbcL	TopHitPlus	Cotoneaster rehderi	Wild	Crataegus	100.000	80
JQ391135.1	rbcL	TopHitPlus	Cotoneaster salicifolius	Wild	Crataegus	100.000	80
KC251251.1	rbcL	TopHitPlus	Crataegus cupressocollina	Absent	Cotoneaster	100.000	60
KC251269.1	rbcL	TopHitPlus	Crataegus douglasii	Absent	Cotoneaster	100.000	60
KC251280.1	rbcL	TopHitPlus	Crataegus enderbyensis	Absent	Cotoneaster	100.000	60
KC251288.1	rbcL	TopHitPlus	Crataegus gaylussacia	Absent	Cotoneaster	99.819	60
MH657670.1	rbcL	TopHitPlus	Crataegus kansuensis	Absent	Amelanchier	100.000	30
KJ506867.1	rbcL	TopHitPlus	Crataegus laevigata	Wild	Amelanchier	99.857	40
MK525625.1	rbcL	TopHitPlus	Crataegus mollis	Absent	Chaenomeles	100.000	40
FN689370.1	rbcL	TopHitPlus	Crataegus monogyna	Wild	Amelanchier	100.000	40
HE963432.1	rbcL	TopHitPlus	Crataegus monogyna	Wild	Cotoneaster	100.000	60
HG765050.1	rbcL	TopHitPlus	Crataegus monogyna	Wild	Amelanchier	100.000	50
HG765052.1	rbcL	TopHitPlus	Crataegus monogyna	Wild	Sorbus	100.000	30
HQ619776.1	rbcL	TopHitPlus	Crataegus monogyna	Wild	Cotoneaster	100.000	60
KC251352.1	rbcL	TopHitPlus	Crataegus monogyna	Wild	Cotoneaster	100.000	80
KJ746209.1	rbcL	TopHitPlus	Crataegus monogyna	Wild	Amelanchier	100.000	50
KM360737.1	rbcL	TopHitPlus	Crataegus monogyna	Wild	Amelanchier	99.857	70
KX162922.1	rbcL	TopHitPlus	Crataegus monogyna	Wild	Amelanchier	99.833	30
MN662636.1	rbcL	TopHitPlus	Crataegus monogyna	Wild	Chaenomeles	100.000	30
KP050248.1	rbcL	TopHitPlus	Crataegus okanaganensis	Absent	Cotoneaster	100.000	70
KC251356.1	rbcL	TopHitPlus	Crataegus okennonii	Absent	Cotoneaster	100.000	80
MK525627.1	rbcL	TopHitPlus	Crataegus pedicellata	Introduced	Chaenomeles	100.000	40
KC251404.1	rbcL	TopHitPlus	Crataegus spathulata	Absent	Cotoneaster	99.638	70
JQ391147.1	rbcL	TopHitPlus	Cydonia oblonga	Introduced	Crataegus	100.000	80
JQ391332.1	rbcL	TopHitPlus	Cydonia oblonga	Introduced	Crataegus	100.000	50
JQ391333.1	rbcL	TopHitPlus	Cydonia oblonga	Introduced	Crataegus	100.000	60
KX499857.1	rbcL	TopHitPlus	Cydonia oblonga	Introduced	Crataegus	99.930	30
JQ391176.1	rbcL	TopHitPlus	Malus baccata	Introduced	Crataegus	100.000	80
JQ391187.1	rbcL	TopHitPlus	Malus ioensis	Absent	Crataegus	100.000	100
JQ391175.1	rbcL	TopHitPlus	Malus mandshurica	Absent	Crataegus	100.000	80
JQ391185.1	rbcL	TopHitPlus	Malus sieboldii	Introduced	Crataegus	100.000	90
JQ391200.1	rbcL	TopHitPlus	Malus sieversii	Absent	Crataegus	100.000	80
JQ391194.1	rbcL	TopHitPlus	Malus sylvestris	Wild	Crataegus	100.000	80
JQ391172.1	rbcL	TopHitPlus	Malus zhaojiaoensis	Absent	Crataegus	100.000	80
JQ391239.1	rbcL	TopHitPlus	Mespilus germanica	Wild	Crataegus	100.000	80
KC206626.1	rbcL	TopHitPlus	Mespilus germanica	Wild	Crataegus	100.000	100
JQ391228.1	rbcL	TopHitPlus	Photinia villosa	Introduced	Crataegus	99.441	100
JQ391207.1	rbcL	TopHitPlus	Pyracantha coccinea	Introduced	Crataegus	100.000	80

TaxID_query	Barcode	Method	Species_true	Wild	Genus	g_Ident	g_Cons
JQ391213.1	rbcL	TopHitPlus	Pyrus communis	Introduced	Crataegus	100.000	80
JQ391209.1	rbcL	TopHitPlus	Pyrus salicifolia	Introduced	Crataegus	100.000	80
JQ391231.1	rbcL	TopHitPlus	Stranvaesia davidiana	Introduced	Crataegus	100.000	80

Idem for Prunus

TaxID_query	Barcode	Method	Species_true	Wild	Genus	g_Ident	g_Cons
MG235427.1	ITS2	TopHitPlus	Rhodotypos scandens	Introduced	Prunus	90.746	90
GQ434215.1	ITS2	TopHitPlus	Sorbaria sorbifolia	Wild	Prunus	94.641	90
GQ435278.1	ITS2	TopHitPlus	Sorbaria sorbifolia	Wild	Prunus	98.947	60
GQ435718.1	ITS2	TopHitPlus	Sorbaria sorbifolia	Wild	Prunus	97.826	50
MK526455.1	rbcL	TopHitPlus	Prunus tomentosa	Introduced	Rosa	100.000	100
KR529964.1	rbcL	TopHitPlus	Pygeum macrocarpum	Absent	Prunus	100.000	90
KF154892.1	rbcL	TopHitPlus	Pygeum topengii	Absent	Prunus	99.901	100

What is the quality of the ID to species level within the genus Prunus ?

We compute the % of correctly identified sequences for the species : “Prunus avium”, “Prunus spinosa”, “Prunus serotina”, “Prunus padus”, “Prunus mahaleb” ,“Prunus cerasus”, “Prunus cerasifera”, “Prunus domestica”

CV_method	DB_gene	Method	Barcode	DB_area	Taxon	R	P	F
10 Fold CV	General	TopHitPlus	ITS2	Local DB	Prunus mahaleb	60.00	100.00	75.00
10 Fold CV	General	TopHitPlus	ITS2	Local DB	Prunus avium	92.31	48.00	63.16
10 Fold CV	General	TopHitPlus	ITS2	Local DB	Prunus padus	50.00	33.33	40.00
10 Fold CV	General	TopHitPlus	ITS2	Local DB	Prunus domestica	40.00	28.57	33.33
10 Fold CV	General	TopHitPlus	ITS2	Local DB	Prunus spinosa	50.00	25.00	33.33
10 Fold CV	General	TopHitPlus	ITS2	Local DB	Prunus cerasifera	33.33	28.57	30.77
10 Fold CV	General	TopHitPlus	ITS2	Local DB	Prunus serotina	33.33	20.00	25.00
10 Fold CV	General	TopHitPlus	ITS2	Local DB	Prunus cerasus	0.00	0.00	NA
10 Fold CV	General	TopHitPlus	ITS2	World DB	Prunus avium	76.92	76.92	76.92
10 Fold CV	General	TopHitPlus	ITS2	World DB	Prunus mahaleb	60.00	100.00	75.00
10 Fold CV	General	TopHitPlus	ITS2	World DB	Prunus padus	50.00	50.00	50.00
10 Fold CV	General	TopHitPlus	ITS2	World DB	Prunus spinosa	50.00	40.00	44.44
10 Fold CV	General	TopHitPlus	ITS2	World DB	Prunus serotina	33.33	50.00	40.00
10 Fold CV	General	TopHitPlus	ITS2	World DB	Prunus cerasifera	16.67	50.00	25.00
10 Fold CV	General	TopHitPlus	ITS2	World DB	Prunus cerasus	0.00	0.00	NA
10 Fold CV	General	TopHitPlus	ITS2	World DB	Prunus domestica	0.00	0.00	NA
10 Fold CV	General	TopHitPlus	rbcL	Local DB	Prunus serotina	84.62	73.33	78.57
10 Fold CV	General	TopHitPlus	rbcL	Local DB	Prunus spinosa	47.06	80.00	59.26
10 Fold CV	General	TopHitPlus	rbcL	Local DB	Prunus cerasus	33.33	100.00	50.00
10 Fold CV	General	TopHitPlus	rbcL	Local DB	Prunus cerasifera	66.67	36.36	47.06
10 Fold CV	General	TopHitPlus	rbcL	Local DB	Prunus mahaleb	25.00	100.00	40.00
10 Fold CV	General	TopHitPlus	rbcL	Local DB	Prunus domestica	41.67	31.25	35.72
10 Fold CV	General	TopHitPlus	rbcL	Local DB	Prunus padus	60.00	21.43	31.58
10 Fold CV	General	TopHitPlus	rbcL	Local DB	Prunus avium	12.50	100.00	22.22
10 Fold CV	General	TopHitPlus	rbcL	World DB	Prunus serotina	84.62	84.62	84.62
10 Fold CV	General	TopHitPlus	rbcL	World DB	Prunus spinosa	47.06	88.89	61.54
10 Fold CV	General	TopHitPlus	rbcL	World DB	Prunus cerasus	33.33	100.00	50.00
10 Fold CV	General	TopHitPlus	rbcL	World DB	Prunus cerasifera	58.33	36.84	45.16
10 Fold CV	General	TopHitPlus	rbcL	World DB	Prunus mahaleb	25.00	100.00	40.00
10 Fold CV	General	TopHitPlus	rbcL	World DB	Prunus domestica	33.33	30.77	32.00
10 Fold CV	General	TopHitPlus	rbcL	World DB	Prunus padus	20.00	16.67	18.18
10 Fold CV	General	TopHitPlus	rbcL	World DB	Prunus avium	0.00	NA	NA

All cases with a wrong species level identification for these species

–> most confusions are between species of the genus Prunus…

TaxID_query	Barcode	Method	Species_true	Wild	Species	s_Ident	s_Cons
FJ899097.1	ITS2	TopHitPlus	Prunus avium	Wild	Prunus pseudocerasus	99.266	90
HQ332167.1	ITS2	TopHitPlus	Prunus avium	Wild	Prunus pseudocerasus	99.429	100
MN644243.1	ITS2	TopHitPlus	Prunus avium	Wild	Prunus serrulata	99.833	40
AF318755.1	ITS2	TopHitPlus	Prunus cerasifera	Wild	Prunus spinosa	99.396	10
GQ434216.1	ITS2	TopHitPlus	Prunus cerasifera	Wild	Prunus glandulosa	99.476	10
GQ436141.1	ITS2	TopHitPlus	Prunus cerasifera	Wild	Prunus persica	99.795	20
GQ436612.1	ITS2	TopHitPlus	Prunus cerasifera	Wild	Prunus armeniaca	99.573	10
U16200.1	ITS2	TopHitPlus	Prunus cerasifera	Wild	Prunus bokhariensis	97.967	20
AF318729.1	ITS2	TopHitPlus	Prunus cerasus	Wild	Prunus avium	99.238	40
EF211080.1	ITS2	TopHitPlus	Prunus cerasus	Wild	Prunus tomentosa	99.020	30
MG237696.1	ITS2	TopHitPlus	Prunus cerasus	Wild	Prunus domestica	99.644	10
AF318713.1	ITS2	TopHitPlus	Prunus domestica	Introduced	Prunus spinosa	99.095	20
HF969272.1	ITS2	TopHitPlus	Prunus domestica	Introduced	Prunus simonii	99.680	10
HF969273.1	ITS2	TopHitPlus	Prunus domestica	Introduced	Prunus salicina	99.522	30
KF718374.1	ITS2	TopHitPlus	Prunus domestica	Introduced	Prunus spinosa	99.150	20
KF718376.1	ITS2	TopHitPlus	Prunus domestica	Introduced	Prunus armeniaca	99.740	20
AF318747.1	ITS2	TopHitPlus	Prunus mahaleb	Wild	Prunus sp.	99.541	10
MG237822.1	ITS2	TopHitPlus	Prunus mahaleb	Wild	Prunus avium	99.771	10
AF318726.1	ITS2	TopHitPlus	Prunus padus	Wild	Prunus virginiana	98.628	10
MG236987.1	ITS2	TopHitPlus	Prunus padus	Wild	Prunus virginiana	99.312	20
MG236609.1	ITS2	TopHitPlus	Prunus persica	Introduced	Prunus serotina	99.696	30
EF211077.1	ITS2	TopHitPlus	Prunus salicina	Absent	Prunus domestica	100.000	20
HM453949.1	ITS2	TopHitPlus	Prunus serotina	Wild	Prunus pseudocerasus	94.706	80
MG236869.1	ITS2	TopHitPlus	Prunus serotina	Wild	Prunus persica	99.696	10
KJ649387.1	ITS2	TopHitPlus	Prunus serrulata	Introduced	Prunus avium	99.833	10
AF318730.1	ITS2	TopHitPlus	Prunus spinosa	Wild	Prunus cerasifera	99.396	10
KF718368.1	ITS2	TopHitPlus	Prunus spinosa	Wild	Prunus domestica	100.000	20
MN722063.1	ITS2	TopHitPlus	Prunus tomentosa	Introduced	Prunus cerasus	99.020	10
AF318742.1	ITS2	TopHitPlus	Prunus virginiana	Introduced	Prunus padus	98.628	10
MG236789.1	ITS2	TopHitPlus	Prunus virginiana	Introduced	Prunus padus	99.312	10
HQ235388.1	rbcL	TopHitPlus	Prunus armeniaca	Introduced	Prunus domestica	99.826	30
HQ235390.1	rbcL	TopHitPlus	Prunus armeniaca	Introduced	Prunus cerasifera	99.826	10
HQ235392.1	rbcL	TopHitPlus	Prunus armeniaca	Introduced	Prunus cerasifera	100.000	30
HE963614.1	rbcL	TopHitPlus	Prunus avium	Wild	Prunus apetala	99.828	10
HQ235393.1	rbcL	TopHitPlus	Prunus avium	Wild	Prunus apetala	100.000	10
JN891862.1	rbcL	TopHitPlus	Prunus avium	Wild	Prunus apetala	100.000	10
JN892271.1	rbcL	TopHitPlus	Prunus avium	Wild	Prunus apetala	100.000	10
KP402609.1	rbcL	TopHitPlus	Prunus avium	Wild	Prunus dielsiana	100.000	10
KP402674.1	rbcL	TopHitPlus	Prunus avium	Wild	Prunus apetala	100.000	10
LT996901.1	rbcL	TopHitPlus	Prunus avium	Wild	Prunus apetala	100.000	10
MG246371.1	rbcL	TopHitPlus	Prunus avium	Wild	Prunus apetala	100.000	10
MH116274.1	rbcL	TopHitPlus	Prunus brachypoda	Absent	Prunus padus	100.000	20
AF227900.1	rbcL	TopHitPlus	Prunus cerasifera	Wild	Prunus domestica	99.662	30
HQ235407.1	rbcL	TopHitPlus	Prunus cerasifera	Wild	Prunus domestica	100.000	30
HQ619778.1	rbcL	TopHitPlus	Prunus cerasifera	Wild	Prunus armeniaca	100.000	20
MN418903.1	rbcL	TopHitPlus	Prunus cerasifera	Wild	Prunus domestica	99.930	10
MT901728.1	rbcL	TopHitPlus	Prunus cerasifera	Wild	Prunus salicina	100.000	10
HQ235415.1	rbcL	TopHitPlus	Prunus cerasus	Wild	Prunus fruticosa	100.000	10
HQ235416.1	rbcL	TopHitPlus	Prunus cerasus	Wild	Prunus apetala	100.000	10
HE963617.1	rbcL	TopHitPlus	Prunus domestica	Introduced	Prunus cerasifera	100.000	50
HQ235427.1	rbcL	TopHitPlus	Prunus domestica	Introduced	Prunus cerasifera	100.000	20
HQ235431.1	rbcL	TopHitPlus	Prunus domestica	Introduced	Prunus cerasifera	99.826	30
JN893011.1	rbcL	TopHitPlus	Prunus domestica	Introduced	Prunus cerasifera	100.000	30
KX163003.1	rbcL	TopHitPlus	Prunus domestica	Introduced	Prunus cerasifera	100.000	30
L01947.2	rbcL	TopHitPlus	Prunus domestica	Introduced	Prunus pedunculata	100.000	20
MG249824.1	rbcL	TopHitPlus	Prunus domestica	Introduced	Prunus cerasifera	100.000	40
MT302569.1	rbcL	TopHitPlus	Prunus domestica	Introduced	Prunus cerasifera	99.930	20
AF411505.1	rbcL	TopHitPlus	Prunus grayana	Absent	Prunus padus	98.999	20
HQ235465.1	rbcL	TopHitPlus	Prunus laurocerasus	Wild	Prunus padus	99.826	20
HE963619.1	rbcL	TopHitPlus	Prunus mahaleb	Wild	Prunus armeniaca	99.314	10
HQ235468.1	rbcL	TopHitPlus	Prunus mahaleb	Wild	Prunus apetala	99.826	10
MN662660.1	rbcL	TopHitPlus	Prunus mahaleb	Wild	Prunus rufa	99.810	10
AF411485.1	rbcL	TopHitPlus	Prunus padus	Wild	Prunus grayana	98.999	10
GU363791.1	rbcL	TopHitPlus	Prunus padus	Wild	Prunus hypoxantha	99.858	10
HQ235499.1	rbcL	TopHitPlus	Prunus padus	Wild	Prunus zippeliana	100.000	30
JN892373.1	rbcL	TopHitPlus	Prunus padus	Wild	Prunus africana	100.000	20
KP088764.1	rbcL	TopHitPlus	Prunus padus	Wild	Prunus sp.	100.000	20
KP760072.1	rbcL	TopHitPlus	Prunus padus	Wild	Prunus hypoxantha	99.790	10
MH657250.1	rbcL	TopHitPlus	Prunus padus	Wild	Prunus zippeliana	100.000	30
MH657643.1	rbcL	TopHitPlus	Prunus padus	Wild	Prunus zippeliana	100.000	30
KJ593639.1	rbcL	TopHitPlus	Prunus pensylvanica	Absent	Prunus serotina	99.815	70

TaxID_query	Barcode	Method	Species_true	Wild	Species	s_Ident	s_Cons
MG248419.1	rbcL	TopHitPlus	Prunus persica	Introduced	Prunus serotina	100.000	50
MF374324.1	rbcL	TopHitPlus	Prunus serotina	Wild	Prunus napaulensis	99.650	10
MK526454.1	rbcL	TopHitPlus	Prunus serotina	Wild	Prunus zippeliana	100.000	20
AF227904.1	rbcL	TopHitPlus	Prunus spinosa	Wild	Prunus domestica	100.000	40
FN689384.1	rbcL	TopHitPlus	Prunus spinosa	Wild	Prunus domestica	99.865	10
HQ235560.1	rbcL	TopHitPlus	Prunus spinosa	Wild	Prunus domestica	100.000	30
JN893265.1	rbcL	TopHitPlus	Prunus spinosa	Wild	Prunus domestica	100.000	40
KJ204394.1	rbcL	TopHitPlus	Prunus spinosa	Wild	Prunus cerasifera	100.000	40
KM360942.1	rbcL	TopHitPlus	Prunus spinosa	Wild	Prunus domestica	99.929	20
KX163006.1	rbcL	TopHitPlus	Prunus spinosa	Wild	Prunus cerasifera	99.666	20
KX163010.1	rbcL	TopHitPlus	Prunus spinosa	Wild	Prunus cerasifera	100.000	30
KY420008.1	rbcL	TopHitPlus	Prunus spinosa	Wild	Prunus undulata	99.370	10
GQ248686.1	rbcL	TopHitPlus	Prunus virginiana	Introduced	Prunus padus	100.000	30
HQ590226.1	rbcL	TopHitPlus	Prunus virginiana	Introduced	Prunus padus	100.000	20
HQ590227.1	rbcL	TopHitPlus	Prunus virginiana	Introduced	Prunus padus	100.000	20
KJ593642.1	rbcL	TopHitPlus	Prunus virginiana	Introduced	Prunus padus	100.000	20
KJ593643.1	rbcL	TopHitPlus	Prunus virginiana	Introduced	Prunus padus	100.000	20
MG247716.1	rbcL	TopHitPlus	Prunus virginiana	Introduced	Prunus padus	100.000	30
MK526456.1	rbcL	TopHitPlus	Prunus virginiana	Introduced	Prunus padus	100.000	20
KR529964.1	rbcL	TopHitPlus	Pygeum macrocarpum	Absent	Prunus spinosa	100.000	10

5.2.2 Brassicaceae

% of correctly identified sequences for the following Brassicaceae Genus (as Target of matched sequences)

```
##  [1] "Brassica"    "Sisymbrium"  "Raphanus"    "Eruca"       "Arabidopsis" "Capsella"
##  [7] "Lepidium"    "Cardamine"   "Rorippa"     "Nasturtium"  "Sinapis"     "Alliaria"
## [13] "Arabis"      "Barbarea"    "Diplotaxis"  "Hesperis"    "Biscutella"  "Thlaspi"
## [19] "Lunaria"     "Teesdalia"
```

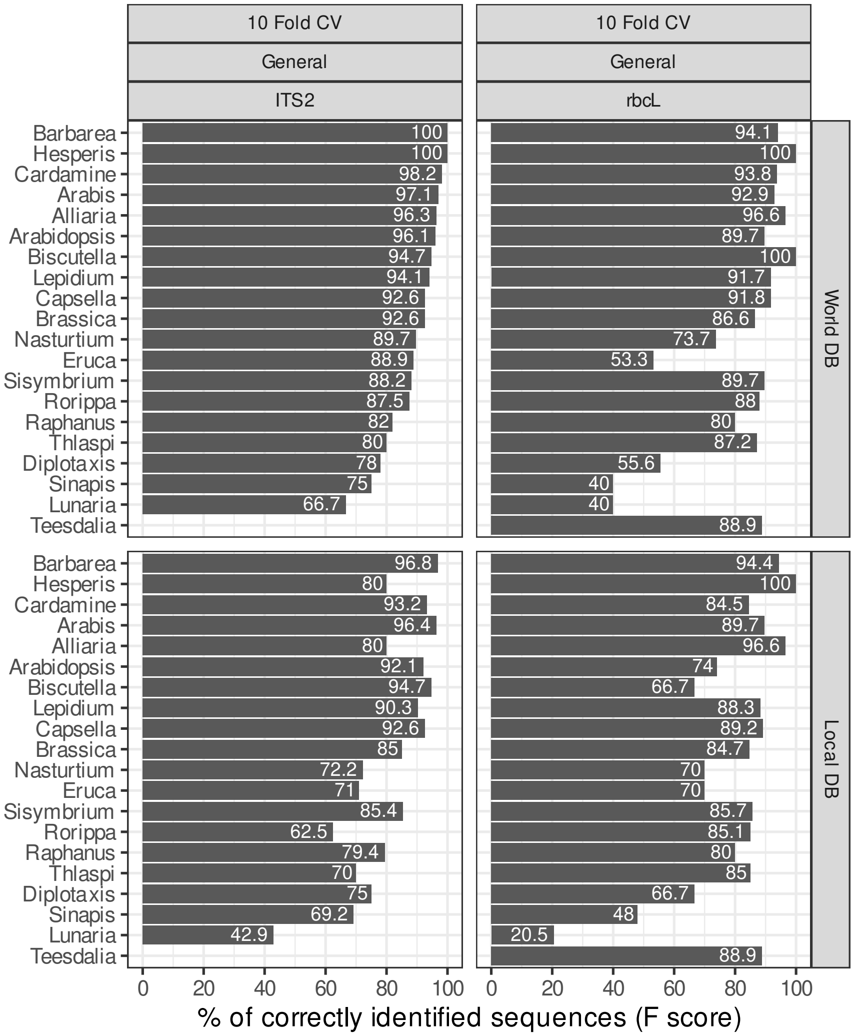

Figure 40:

CV_method	DB_gene	Method	Barcode	DB_area	Taxon	R	P	F
10 Fold CV	General	TopHitPlus	ITS2	World DB	Barbarea	100.00	100.00	100.00
10 Fold CV	General	TopHitPlus	ITS2	World DB	Hesperis	100.00	100.00	100.00
10 Fold CV	General	TopHitPlus	ITS2	World DB	Cardamine	97.72	98.62	98.17
10 Fold CV	General	TopHitPlus	ITS2	World DB	Arabis	98.81	95.40	97.08
10 Fold CV	General	TopHitPlus	ITS2	World DB	Alliaria	92.86	100.00	96.30
10 Fold CV	General	TopHitPlus	ITS2	World DB	Arabidopsis	96.52	95.69	96.10
10 Fold CV	General	TopHitPlus	ITS2	World DB	Biscutella	90.00	100.00	94.74
10 Fold CV	General	TopHitPlus	ITS2	World DB	Lepidium	94.74	93.51	94.12
10 Fold CV	General	TopHitPlus	ITS2	World DB	Capsella	92.59	92.59	92.59
10 Fold CV	General	TopHitPlus	ITS2	World DB	Brassica	94.57	90.62	92.55
10 Fold CV	General	TopHitPlus	ITS2	World DB	Nasturtium	86.67	92.86	89.66
10 Fold CV	General	TopHitPlus	ITS2	World DB	Eruca	92.31	85.71	88.89
10 Fold CV	General	TopHitPlus	ITS2	World DB	Sisymbrium	86.54	90.00	88.24

CV_method	DB_gene	Method	Barcode	DB_area	Taxon	R	P	F
10 Fold CV	General	TopHitPlus	ITS2	World DB	Rorippa	87.50	87.50	87.50
10 Fold CV	General	TopHitPlus	ITS2	World DB	Raphanus	86.21	78.12	81.97
10 Fold CV	General	TopHitPlus	ITS2	World DB	Thlaspi	73.68	87.50	80.00
10 Fold CV	General	TopHitPlus	ITS2	World DB	Diplotaxis	72.73	84.21	78.05
10 Fold CV	General	TopHitPlus	ITS2	World DB	Sinapis	69.23	81.82	75.00
10 Fold CV	General	TopHitPlus	ITS2	World DB	Lunaria	60.00	75.00	66.67
10 Fold CV	General	TopHitPlus	ITS2	World DB	Teesdalia	0.00	NA	NA
10 Fold CV	General	TopHitPlus	ITS2	Local DB	Barbarea	100.00	93.75	96.77
10 Fold CV	General	TopHitPlus	ITS2	Local DB	Arabis	95.24	97.56	96.39
10 Fold CV	General	TopHitPlus	ITS2	Local DB	Biscutella	90.00	100.00	94.74
10 Fold CV	General	TopHitPlus	ITS2	Local DB	Cardamine	97.26	89.50	93.22
10 Fold CV	General	TopHitPlus	ITS2	Local DB	Capsella	92.59	92.59	92.59
10 Fold CV	General	TopHitPlus	ITS2	Local DB	Arabidopsis	96.52	88.10	92.12
10 Fold CV	General	TopHitPlus	ITS2	Local DB	Lepidium	92.11	88.61	90.33
10 Fold CV	General	TopHitPlus	ITS2	Local DB	Sisymbrium	90.38	81.03	85.45
10 Fold CV	General	TopHitPlus	ITS2	Local DB	Brassica	95.11	76.75	84.95
10 Fold CV	General	TopHitPlus	ITS2	Local DB	Alliaria	100.00	66.67	80.00
10 Fold CV	General	TopHitPlus	ITS2	Local DB	Hesperis	85.71	75.00	80.00
10 Fold CV	General	TopHitPlus	ITS2	Local DB	Raphanus	86.21	73.53	79.37
10 Fold CV	General	TopHitPlus	ITS2	Local DB	Diplotaxis	68.18	83.33	75.00
10 Fold CV	General	TopHitPlus	ITS2	Local DB	Nasturtium	86.67	61.90	72.22
10 Fold CV	General	TopHitPlus	ITS2	Local DB	Eruca	84.62	61.11	70.97
10 Fold CV	General	TopHitPlus	ITS2	Local DB	Thlaspi	73.68	66.67	70.00
10 Fold CV	General	TopHitPlus	ITS2	Local DB	Sinapis	69.23	69.23	69.23
10 Fold CV	General	TopHitPlus	ITS2	Local DB	Rorippa	62.50	62.50	62.50
10 Fold CV	General	TopHitPlus	ITS2	Local DB	Lunaria	60.00	33.33	42.85
10 Fold CV	General	TopHitPlus	ITS2	Local DB	Teesdalia	0.00	0.00	NA
10 Fold CV	General	TopHitPlus	rbcL	World DB	Biscutella	100.00	100.00	100.00
10 Fold CV	General	TopHitPlus	rbcL	World DB	Hesperis	100.00	100.00	100.00
10 Fold CV	General	TopHitPlus	rbcL	World DB	Alliaria	100.00	93.33	96.55
10 Fold CV	General	TopHitPlus	rbcL	World DB	Barbarea	88.89	100.00	94.12
10 Fold CV	General	TopHitPlus	rbcL	World DB	Cardamine	95.24	92.31	93.75
10 Fold CV	General	TopHitPlus	rbcL	World DB	Arabis	86.67	100.00	92.86
10 Fold CV	General	TopHitPlus	rbcL	World DB	Capsella	90.32	93.33	91.80
10 Fold CV	General	TopHitPlus	rbcL	World DB	Lepidium	91.67	91.67	91.67
10 Fold CV	General	TopHitPlus	rbcL	World DB	Arabidopsis	92.86	86.67	89.66
10 Fold CV	General	TopHitPlus	rbcL	World DB	Sisymbrium	89.66	89.66	89.66
10 Fold CV	General	TopHitPlus	rbcL	World DB	Teesdalia	80.00	100.00	88.89
10 Fold CV	General	TopHitPlus	rbcL	World DB	Rorippa	91.67	84.62	88.00
10 Fold CV	General	TopHitPlus	rbcL	World DB	Thlaspi	85.00	89.47	87.18
10 Fold CV	General	TopHitPlus	rbcL	World DB	Brassica	85.29	87.88	86.57
10 Fold CV	General	TopHitPlus	rbcL	World DB	Raphanus	70.59	92.31	80.00
10 Fold CV	General	TopHitPlus	rbcL	World DB	Nasturtium	63.64	87.50	73.69
10 Fold CV	General	TopHitPlus	rbcL	World DB	Diplotaxis	62.50	50.00	55.56
10 Fold CV	General	TopHitPlus	rbcL	World DB	Eruca	44.44	66.67	53.33
10 Fold CV	General	TopHitPlus	rbcL	World DB	Lunaria	25.00	100.00	40.00
10 Fold CV	General	TopHitPlus	rbcL	World DB	Sinapis	42.11	38.10	40.00
10 Fold CV	General	TopHitPlus	rbcL	Local DB	Hesperis	100.00	100.00	100.00
10 Fold CV	General	TopHitPlus	rbcL	Local DB	Alliaria	100.00	93.33	96.55
10 Fold CV	General	TopHitPlus	rbcL	Local DB	Barbarea	94.44	94.44	94.44
10 Fold CV	General	TopHitPlus	rbcL	Local DB	Arabis	86.67	92.86	89.66
10 Fold CV	General	TopHitPlus	rbcL	Local DB	Capsella	93.55	85.29	89.23
10 Fold CV	General	TopHitPlus	rbcL	Local DB	Teesdalia	80.00	100.00	88.89
10 Fold CV	General	TopHitPlus	rbcL	Local DB	Lepidium	88.33	88.33	88.33
10 Fold CV	General	TopHitPlus	rbcL	Local DB	Sisymbrium	93.10	79.41	85.71
10 Fold CV	General	TopHitPlus	rbcL	Local DB	Rorippa	83.33	86.96	85.11
10 Fold CV	General	TopHitPlus	rbcL	Local DB	Thlaspi	85.00	85.00	85.00
10 Fold CV	General	TopHitPlus	rbcL	Local DB	Brassica	85.29	84.06	84.67
10 Fold CV	General	TopHitPlus	rbcL	Local DB	Cardamine	95.24	75.95	84.51
10 Fold CV	General	TopHitPlus	rbcL	Local DB	Raphanus	70.59	92.31	80.00
10 Fold CV	General	TopHitPlus	rbcL	Local DB	Arabidopsis	96.43	60.00	73.97
10 Fold CV	General	TopHitPlus	rbcL	Local DB	Eruca	77.78	63.64	70.00
10 Fold CV	General	TopHitPlus	rbcL	Local DB	Nasturtium	63.64	77.78	70.00
10 Fold CV	General	TopHitPlus	rbcL	Local DB	Biscutella	66.67	66.67	66.67
10 Fold CV	General	TopHitPlus	rbcL	Local DB	Diplotaxis	62.50	71.43	66.67
10 Fold CV	General	TopHitPlus	rbcL	Local DB	Sinapis	63.16	38.71	48.00
10 Fold CV	General	TopHitPlus	rbcL	Local DB	Lunaria	100.00	11.43	20.52

Wrong genus level identification for Brassica and Sinapis –> often confounded with each other and Raphanus but less with other Wild Brassicaceae

TaxID_query	Barcode	Method	Species_true	Wild	Genus	g_Ident	g_Cons
AY722417.1	ITS2	TopHitPlus	Brassica elongata	Introduced	Erucastrum	99.643	10.00
GQ268078.1	ITS2	TopHitPlus	Brassica elongata	Introduced	Erucastrum	99.642	10.00
GQ435335.1	ITS2	TopHitPlus	Brassica juncea	Introduced	Raphanus	81.102	50.00
GQ435757.1	ITS2	TopHitPlus	Brassica juncea	Introduced	Isatis	97.662	10.00
GQ436184.1	ITS2	TopHitPlus	Brassica juncea	Introduced	Raphanus	99.241	10.00
GQ436652.1	ITS2	TopHitPlus	Brassica juncea	Introduced	Raphanus	99.573	10.00
GQ436653.1	ITS2	TopHitPlus	Brassica juncea	Introduced	Raphanus	99.431	10.00
KM892646.1	ITS2	TopHitPlus	Brassica juncea	Introduced	Lepidium	100.000	100.00
MG234631.1	ITS2	TopHitPlus	Brassica nigra	Wild	Sinapis	100.000	10.00
KX282043.1	ITS2	TopHitPlus	Brassica tournefortii	Introduced	Raphanus	97.374	40.00
MG235121.1	ITS2	TopHitPlus	Conringia orientalis	Introduced	Brassica	100.000	100.00
AY722463.1	ITS2	TopHitPlus	Erucastrum littoreum	Absent	Brassica	97.857	80.00
AY722468.1	ITS2	TopHitPlus	Guiraoa arvensis	Absent	Brassica	95.752	100.00
LN589687.1	ITS2	TopHitPlus	Hemicrambe fruticulosa	Absent	Brassica	82.709	60.00
AF040032.1	ITS2	TopHitPlus	Hirschfeldia incana	Wild	Brassica	100.000	10.00
AY722470.1	ITS2	TopHitPlus	Hirschfeldia incana	Wild	Sinapis	98.582	10.00
DQ249820.1	ITS2	TopHitPlus	Hirschfeldia incana	Wild	Brassica	100.000	10.00
GQ435755.1	ITS2	TopHitPlus	Isatis tinctoria	Introduced	Brassica	97.662	10.00
GQ424548.1	ITS2	TopHitPlus	Nasturtiopsis coronopifolia	Absent	Brassica	90.762	90.00
GQ435337.1	ITS2	TopHitPlus	Raphanus sativus	Introduced	Brassica	81.102	14.29
GQ436185.1	ITS2	TopHitPlus	Raphanus sativus	Introduced	Brassica	99.241	10.00
GQ436654.1	ITS2	TopHitPlus	Raphanus sativus	Introduced	Brassica	99.573	20.00
AY722487.1	ITS2	TopHitPlus	Sinapis arvensis	Wild	Brassica	98.053	100.00
KY968829.1	ITS2	TopHitPlus	Sinapis arvensis	Wild	Brassica	94.875	100.00
KY968908.1	ITS2	TopHitPlus	Sinapis arvensis	Wild	Brassica	98.684	100.00
MG235170.1	ITS2	TopHitPlus	Sinapis arvensis	Wild	Brassica	100.000	100.00
MG236132.1	ITS2	TopHitPlus	Sisymbrium altissimum	Wild	Brassica	96.882	100.00
MG236819.1	ITS2	TopHitPlus	Sisymbrium altissimum	Wild	Brassica	97.115	70.00
AB856329.1	ITS2	TopHitPlus	Sisymbrium orientale	Wild	Brassica	96.312	100.00
KX824594.1	rbcL	TopHitPlus	Brassica juncea	Introduced	Draba	100.000	100.00
HM849822.1	rbcL	TopHitPlus	Brassica nigra	Wild	Hirschfeldia	99.853	10.00
JN891537.1	rbcL	TopHitPlus	Brassica nigra	Wild	Coincya	100.000	40.00
JN893339.1	rbcL	TopHitPlus	Brassica nigra	Wild	Cakile	100.000	30.00
MG245964.1	rbcL	TopHitPlus	Brassica nigra	Wild	Sinapis	100.000	40.00
MG247478.1	rbcL	TopHitPlus	Brassica nigra	Wild	Sinapis	100.000	40.00
AB711140.1	rbcL	TopHitPlus	Brassica oleracea	Introduced	Erucastrum	100.000	30.00
KX282607.1	rbcL	TopHitPlus	Brassica tournefortii	Introduced	Sinapis	100.000	50.00
KX282608.1	rbcL	TopHitPlus	Brassica tournefortii	Introduced	Sinapis	100.000	50.00
KX282609.1	rbcL	TopHitPlus	Brassica tournefortii	Introduced	Sinapis	100.000	50.00
JN891266.1	rbcL	TopHitPlus	Coincya monensis	Wild	Sinapis	100.000	40.00
MK925074.1	rbcL	TopHitPlus	Coincya wrightii	Absent	Sinapis	100.000	40.00
MG246579.1	rbcL	TopHitPlus	Conringia orientalis	Introduced	Brassica	100.000	100.00
MG248484.1	rbcL	TopHitPlus	Conringia orientalis	Introduced	Brassica	100.000	90.00
JN893186.1	rbcL	TopHitPlus	Crambe maritima	Wild	Sinapis	99.631	50.00
KU739565.1	rbcL	TopHitPlus	Erucastrum gallicum	Introduced	Brassica	99.716	80.00
MK637718.1	rbcL	TopHitPlus	Guiraoa arvensis	Absent	Sinapis	99.861	20.00
KM360819.1	rbcL	TopHitPlus	Hirschfeldia incana	Wild	Sinapis	99.645	20.00
JN891109.1	rbcL	TopHitPlus	Raphanus raphanistrum	Wild	Sinapis	100.000	10.00
KX678762.1	rbcL	TopHitPlus	Raphanus raphanistrum	Wild	Sinapis	100.000	10.00
GQ184381.1	rbcL	TopHitPlus	Raphanus sativus	Introduced	Brassica	99.731	80.00
HM850300.1	rbcL	TopHitPlus	Rapistrum rugosum	Introduced	Brassica	98.095	60.00
KX421123.1	rbcL	TopHitPlus	Rapistrum rugosum	Introduced	Sinapis	99.669	40.00
HM849823.1	rbcL	TopHitPlus	Sinapis alba	Introduced	Moricandia	98.019	10.00
JN892988.1	rbcL	TopHitPlus	Sinapis alba	Introduced	Coincya	100.000	40.00
JN893803.1	rbcL	TopHitPlus	Sinapis alba	Introduced	Cakile	99.252	40.00
MG247630.1	rbcL	TopHitPlus	Sinapis alba	Introduced	Raphanus	100.000	90.00
HQ590272.1	rbcL	TopHitPlus	Sinapis arvensis	Wild	Brassica	100.000	30.00
JN893802.1	rbcL	TopHitPlus	Sinapis arvensis	Wild	Coincya	100.000	40.00
KJ841573.1	rbcL	TopHitPlus	Sinapis arvensis	Wild	Coincya	100.000	30.00
KU050690.1	rbcL	TopHitPlus	Sinapis arvensis	Wild	Brassica	99.931	20.00
MF135323.1	rbcL	TopHitPlus	Sinapis arvensis	Wild	Hirschfeldia	100.000	10.00
MF135409.1	rbcL	TopHitPlus	Sinapis arvensis	Wild	Brassica	100.000	100.00
MK526677.1	rbcL	TopHitPlus	Sinapis arvensis	Wild	Coincya	100.000	30.00

6 Session Info

```
## R version 4.3.1 (2023-06-16)
## Platform: x86_64-pc-linux-gnu (64-bit)
## Running under: Ubuntu 22.04.3 LTS
##
## Matrix products: default
## BLAS:   /usr/lib/x86_64-linux-gnu/openblas-pthread/libblas.so.3
## LAPACK: /usr/lib/x86_64-linux-gnu/openblas-pthread/libopenblasp-r0.3.20.so;  LAPACK version 3.10.0
##
## locale:
##  [1] LC_CTYPE=en_GB.UTF-8       LC_NUMERIC=C               LC_TIME=en_GB.UTF-8
##  [4] LC_COLLATE=en_GB.UTF-8     LC_MONETARY=en_GB.UTF-8    LC_MESSAGES=fr_BE.UTF-8
##  [7] LC_PAPER=fr_BE.UTF-8       LC_NAME=C                  LC_ADDRESS=C
## [10] LC_TELEPHONE=C             LC_MEASUREMENT=fr_BE.UTF-8 LC_IDENTIFICATION=C
##
## time zone: Europe/Brussels
## tzcode source: system (glibc)
##
## attached base packages:
## [1] stats4    grid      stats     graphics  grDevices utils     datasets  methods   base
##
## other attached packages:
##  [1] CVrefDB_0.0.1     dplyr_1.1.2       tidyr_1.3.0       purrr_1.0.2       broom_1.0.5
##  [6] data.table_1.14.8 partykit_1.2-17   libcoin_1.0-9     party_1.3-12      strucchange_1.5-3
## [11] sandwich_3.0-2    zoo_1.8-11        modeltools_0.2-23 mvtnorm_1.1-3     rpart_4.1.19
## [16] ggplot2_3.4.3     pander_0.6.5      knitr_1.43
##
## loaded via a namespace (and not attached):
##  [1] gtable_0.3.4           xfun_0.39              coin_1.4-2
##  [4] lattice_0.21-8         vctrs_0.6.3            tools_4.3.1
##  [7] bitops_1.0-7           generics_0.1.3         parallel_4.3.1
## [10] tibble_3.2.1           fansi_1.0.4            pkgconfig_2.0.3
## [13] Matrix_1.6-0           S4Vectors_0.34.0       lifecycle_1.0.3
## [16] GenomeInfoDbData_1.2.8 farver_2.1.1           compiler_4.3.1
## [19] Biostrings_2.64.1      munsell_0.5.0          codetools_0.2-19
## [22] GenomeInfoDb_1.32.4    htmltools_0.5.5        RCurl_1.98-1.10
## [25] yaml_2.3.7             Formula_1.2-5          pillar_1.9.0
## [28] crayon_1.5.2           MASS_7.3-60            multcomp_1.4-22
## [31] nlme_3.1-162           tidyselect_1.2.0       digest_0.6.31
## [34] inum_1.0-4             bookdown_0.33          labeling_0.4.2
## [37] splines_4.3.1          fastmap_1.1.1          colorspace_2.1-0
## [40] cli_3.6.1              magrittr_2.0.3         survival_3.5-5
## [43] utf8_1.2.3             TH.data_1.1-1          withr_2.5.0
## [46] scales_1.2.1           backports_1.4.1        XVector_0.36.0
## [49] rmarkdown_2.22         matrixStats_0.63.0     gridExtra_2.3
## [52] evaluate_0.21          IRanges_2.30.1         mgcv_1.9-0
## [55] rlang_1.1.1            Rcpp_1.0.10            glue_1.6.2
## [58] pROC_1.18.0            BiocGenerics_0.42.0    rstudioapi_0.14
## [61] plyr_1.8.8             R6_2.5.1               zlibbioc_1.42.0
```

7 References

Automatic citation of R and all packages used :

R
R Core Team (2023). *R: A Language and Environment for Statistical Computing*. R Foundation for Statistical Computing, Vienna, Austria. https://www.R-project.org/.

broom
Robinson D, Hayes A, Couch S (2023). *broom: Convert Statistical Objects into Tidy Tibbles*. R package version 1.0.5, https://CRAN.R-project.org/package=broom.

CVrefDB
San Martin G (2023). *CVrefDB: Cross Validation (CV) of DNA barcodes reference databases (refDB) using blast*. R package version 0.0.1, https://github.com/GillesSanMartin/CVrefDB.

data.table
Dowle M, Srinivasan A (2023). *data.table: Extension of `data.frame`*. R package version 1.14.8, https://CRAN.R-project.org/package=data.table.

dplyr
Wickham H, François R, Henry L, Müller K, Vaughan D (2023). *dplyr: A Grammar of Data Manipulation*. R package version 1.1.2, https://CRAN.R-project.org/package=dplyr.

ggplot2
Wickham H (2016). *ggplot2: Elegant Graphics for Data Analysis*. Springer-Verlag New York. ISBN 978-3-319-24277-4, https://ggplot2.tidyverse.org.

knitr
Xie Y (2023). *knitr: A General-Purpose Package for Dynamic Report Generation in R*. R package version 1.43, https://yihui.org/knitr/.

Xie Y (2015). *Dynamic Documents with R and knitr*, 2nd edition. Chapman and Hall/CRC, Boca Raton, Florida. ISBN 978-1498716963, https://yihui.org/knitr/.

Xie Y (2014). "knitr: A Comprehensive Tool for Reproducible Research in R." In Stodden V, Leisch F, Peng RD (eds.), *Implementing Reproducible Computational Research*. Chapman and Hall/CRC. ISBN 978-1466561595.

libcoin
Hothorn T (2021). *libcoin: Linear Test Statistics for Permutation Inference*. R package version 1.0-9, https://CRAN.R-project.org/package=libcoin.

modeltools
Hothorn T, Leisch F, Zeileis A (2020). *modeltools: Tools and Classes for Statistical Models*. R package version 0.2-23, https://CRAN.R-project.org/package=modeltools.

mvtnorm
Genz A, Bretz F, Miwa T, Mi X, Leisch F, Scheipl F, Hothorn T (2021). *mvtnorm: Multivariate Normal and t Distributions*. R package version 1.1-3, https://CRAN.R-project.org/package=mvtnorm.

Genz A, Bretz F (2009). *Computation of Multivariate Normal and t Probabilities*, series Lecture Notes in Statistics. Springer-Verlag, Heidelberg. ISBN 978-3-642-01688-2.

pander
Daróczi G, Tsegelskyi R (2022). *pander: An R 'Pandoc' Writer*. R package version 0.6.5, https://CRAN.R-project.org/package=pander.

party
Hothorn T, Hornik K, Zeileis A (2006). "Unbiased Recursive Partitioning: A Conditional Inference Framework." *Journal of Computational and Graphical Statistics*, *15*(3), 651-674. doi:10.1198/106186006X133933 https://doi.org/10.1198/106186006X133933.

Zeileis A, Hothorn T, Hornik K (2008). "Model-Based Recursive Partitioning." *Journal of Computational and Graphical Statistics*, *17*(2), 492-514. doi:10.1198/106186008X319331 https://doi.org/10.1198/106186008X319331.

Hothorn T, Buehlmann P, Dudoit S, Molinaro A, Van Der Laan M (2006). "Survival Ensembles." *Biostatistics*, *7*(3), 355-373.

Strobl C, Boulesteix A, Zeileis A, Hothorn T (2007). "Bias in Random Forest Variable Importance Measures: Illustrations, Sources and a Solution." *BMC Bioinformatics*, *8*(25). doi:10.1186/1471-2105-8-25 https://doi.org/10.1186/1471-2105-8-25.

Strobl C, Boulesteix A, Kneib T, Augustin T, Zeileis A (2008). "Conditional Variable Importance for Random Forests." *BMC Bioinformatics*, *9*(307). doi:10.1186/1471-2105-9-307 https://doi.org/10.1186/1471-2105-9-307.

partykit
Hothorn T, Zeileis A (2015). "partykit: A Modular Toolkit for Recursive Partytioning in R." *Journal of Machine Learning Research*, *16*, 3905-3909. https://jmlr.org/papers/v16/hothorn15a.html.

Hothorn T, Hornik K, Zeileis A (2006). "Unbiased Recursive Partitioning: A Conditional Inference Framework." *Journal of Computational and Graphical Statistics*, *15*(3), 651-674. doi:10.1198/106186006X133933 https://doi.org/10.1198/106186006X133933.

Zeileis A, Hothorn T, Hornik K (2008). "Model-Based Recursive Partitioning." *Journal of Computational and Graphical Statistics*, *17*(2), 492-514. doi:10.1198/106186008X319331 https://doi.org/10.1198/106186008X319331.

purrr
Wickham H, Henry L (2023). *purrr: Functional Programming Tools*. R package version 1.0.2, https://CRAN.R-project.org/package=purrr.

rpart
Therneau T, Atkinson B (2022). *rpart: Recursive Partitioning and Regression Trees*. R package version 4.1.19, https://CRAN.R-project.org/package=rpart.

sandwich
Zeileis A, Köll S, Graham N (2020). "Various Versatile Variances: An Object-Oriented Implementation of Clustered Covariances in R." *Journal of Statistical Software*, *95*(1), 1-36. doi:10.18637/jss.v095.i01 https://doi.org/10.18637/jss.v095.i01.

Zeileis A (2004). "Econometric Computing with HC and HAC Covariance Matrix Estimators." *Journal of Statistical Software*, *11*(10), 1-17. doi:10.18637/jss.v011.i10 https://doi.org/10.18637/jss.v011.i10.

Zeileis A (2006). "Object-Oriented Computation of Sandwich Estimators." *Journal of Statistical Software*, *16*(9), 1-16. doi:10.18637/jss.v016.i09 https://doi.org/10.18637/jss.v016.i09.

strucchange
Zeileis A, Leisch F, Hornik K, Kleiber C (2002). "strucchange: An R Package for Testing for Structural Change in Linear Regression Models." *Journal of Statistical Software*, *7*(2), 1-38. doi:10.18637/jss.v007.i02 https://doi.org/10.18637/jss.v007.i02.

Zeileis A, Kleiber C, Krämer W, Hornik K (2003). "Testing and Dating of Structural Changes in Practice." *Computational Statistics & Data Analysis*, *44*(1-2), 109-123. doi:10.1016/S0167-9473(03)00030-6 https://doi.org/10.1016/S0167-9473%2803%2900030-6.

Zeileis A (2006). "Implementing a Class of Structural Change Tests: An Econometric Computing Approach." *Computational Statistics & Data Analysis*, *50*(11), 2987-3008. doi:10.1016/j.csda.2005.07.001 https://doi.org/10.1016/j.csda.2005.07.001.

tidyr
Wickham H, Vaughan D, Girlich M (2023). *tidyr: Tidy Messy Data*. R package version 1.3.0, https://CRAN.R-project.org/package=tidyr.

zoo
Zeileis A, Grothendieck G (2005). "zoo: S3 Infrastructure for Regular and Irregular Time Series." *Journal of Statistical Software*, *14*(6), 1-27. doi:10.18637/jss.v014.i06 https://doi.org/10.18637/jss.v014.i06.
